# Contribution of type W human endogenous retroviruses to the human genome: characterization of HERV-W proviral insertions and processed pseudogenes

**DOI:** 10.1186/s12977-016-0301-x

**Published:** 2016-09-09

**Authors:** Nicole Grandi, Marta Cadeddu, Jonas Blomberg, Enzo Tramontano

**Affiliations:** 1 Department of Life and Environmental Sciences, University of Cagliari, Cittadella Universitaria di Monserrato SS554, 09042 Monserrato, Cagliari, Italy; 2 Department of Medical Sciences, Uppsala University, Uppsala, Sweden; 3 Istituto di Ricerca Genetica e Biomedica, Consiglio Nazionale delle Ricerche (CNR), Monserrato, Cagliari, Italy

**Keywords:** HERV-W, Endogenous retroviruses, HERV, Syncytin

## Abstract

**Background:**

Human endogenous retroviruses (HERVs) are ancient sequences integrated in the germ line cells and vertically transmitted through the offspring constituting about 8 % of our genome. In time, HERVs accumulated mutations that compromised their coding capacity. A prominent exception is HERV-W locus 7q21.2, producing a functional Env protein (Syncytin-1) coopted for placental syncytiotrophoblast formation. While expression of HERV-W sequences has been investigated for their correlation to disease, an exhaustive description of the group composition and characteristics is still not available and current HERV-W group information derive from studies published a few years ago that, of course, used the rough assemblies of the human genome available at that time. This hampers the comparison and correlation with current human genome assemblies.

**Results:**

In the present work we identified and described in detail the distribution and genetic composition of 213 HERV-W elements. The bioinformatics analysis led to the characterization of several previously unreported features and provided a phylogenetic classification of two main subgroups with different age and structural characteristics. New facts on HERV-W genomic context of insertion and co-localization with sequences putatively involved in disease development are also reported.

**Conclusions:**

The present work is a detailed overview of the HERV-W contribution to the human genome and provides a robust genetic background useful to clarify HERV-W role in pathologies with poorly understood etiology, representing, to our knowledge, the most complete and exhaustive HERV-W dataset up to date.

**Electronic supplementary material:**

The online version of this article (doi:10.1186/s12977-016-0301-x) contains supplementary material, which is available to authorized users.

## Background

More than 40 years after the first evidence of discrepancy between the amount of genetic material and organisms complexity, it is now established that less than 2 % of the human genome is composed of protein-coding regions [1]. With respect to this data, it is impressive to consider that human endogenous retroviruses (HERVs) represent four times this value, constituting about the 8 % of our DNA [2]. HERV sequences seem to have been acquired through a traditional infective process, occurred mostly over 30 million years ago [3]. The reverse transcription of the viral genome and the further integration into the germ line cells allowed the Mendelian transmission of these elements through the offspring, determining their coevolution with the host genome.

HERVs belong to class-I transposable elements, termed also retrotransposons, which duplicate through a reverse-transcribed RNA intermediate. Beside HERVs this group comprises also elements devoid of long terminal repeats (LTRs), such as long and short interspersed nuclear elements (LINEs and SINEs respectively). Despite their abundant presence, HERV classification has been for a long time incomplete and sometimes controversial [4], and a comprehensive dataset of the HERV elements present in the human genome has been only recently provided [5]. In particular, HERVs are distributed among three main classes based on sequence similarity with the exogenous members: class I (Gammaretrovirus- and Epsilonretrovirus-like), class II (Betaretrovirus-like) and class III (Spumaretrovirus-like). Each class encloses a variable number of groups [5]. HERV groups have been traditionally identified with a letter according to the type of human tRNA that binds the primer binding site (PBS) during the reverse transcription process [6]. For example, HERV-K elements are supposed to use a Lysine tRNA. Some groups have also been occasionally named according to a neighbor gene (HERV-ADP) or a particular amino acid motif (HERV-FRD). These nomenclatures are now considered inadequate, and taxonomic studies of HERV groups are usually performed using a phylogenetic approach, commonly based on the highly conserved *pol* gene [7]. Currently, HERV primary integrations can be divided into 39 groups, and this panorama is further complicated by 31 additional “non canonical” groups of mosaic forms arisen from secondary integrations or recombination events [5].

For few HERV groups, viral spreading in human chromosomes was not only due to new infections generating novel provirus integrations, but it was also mediated by alternative mechanisms. It is in fact known that several elements of the HERV-W multi-copy group derive from the retrotranscription and mobilization of proviral RNA transcripts mediated by human LINE (L1) machinery, that is responsible for their insertion into new genomic regions. Those sequences are structurally colinear with the proviral mRNA and are called processed pseudogenes [8]. Moreover, the human genome harbors several hundreds of solitary HERV-W LTRs deriving from homologous recombination between the 5′- and 3′ LTRs that removed the retroviral internal part [9].

Regardless of the mechanism of formation, the genomic persistence of HERV sequences during evolution led to the accumulation of several mutations, insertions and deletions, that have generally compromised their coding capacity [10]. A prominent exception is once again represented by the HERV-W group. Initially identified for its possible role in Multiple Sclerosis (MS), this group showed a high expression level in placental tissues. Further investigations interestingly revealed that an HERV-W provirus, named ERVWE1 and localized to locus 7q21.2, (1) retained a complete *env* Open Reading Frame (ORF) [11]; (2) was able to produce a functional protein, called Syncytin-1 and (3) had been co-opted by the human genome for the trophoblast cells fusion during pregnancy, an important structure for regulating the exchanges between mother and fetus [12–14].

Starting from these findings, the expression and coding capacity of HERV-W group have been investigated in the different tissues, above all to find a correlation to various diseases, such as MS [15–21], Schizophrenia [22, 23] and bipolar disorder [24, 25], comprising also a number of pathologies with poorly understood etiology, such as osteoarthritis and cutaneous T cell lymphoma [26, 27]. However, despite the great interest in HERV-W expression, no definitive correlation with human pathologies have been conclusively demonstrated so far [28] and the characterization of the group at the genomic level still remains a major genetic goal and a bioinformatics challenge [29]. Specifically, the current knowledge of the HERV-W genomic distribution and number of copies is still referred to analyses performed a few years ago [8, 30, 31]. In particular, Voisset et al. described the presence of 70, 100, and 30 HERV-W-related *gag*, *pro*, and *env* regions respectively, using a PCR approach on isolated chromosomes DNA samples with HERV-W-specific primers [30]. Costas identified a total of 140 HERV-W elements through a NCBI BLAST within the draft sequence of the human genome [31]. Pavlícek et al. reported 311 HERV-W elements and 343 solitary LTRs identified using the RepeatMasker program in the GoldenPath assembly of 87 % of the human genome [8] These works represent milestones in the HERV-W group characterization, but the absence of a complete and exhaustive version of the human genome and the use of different methodologies make it hard to compare and correlate these data with the current version of the human genome.

Moreover, with the exception of the well-described Syncytin-1 provirus, detailed information about the group composition and its members characteristics are somehow lacking, preventing a comprehensive analysis of their possible involvement in human pathologies. In fact, a detailed knowledge of HERV-W genic origin is essential to complete the previously mentioned observed expression profiles [16–25, 32] and to evaluate their possible involvement in disease development and/or progression. Furthermore, it is well known that the mere presence of HERV integrated elements could affect human physiology and health through alternative mechanisms even in the absence of gene expression or products. This can occur for example (1) with gene physical disruption after HERV insertion [33, 34]; (2) by damaging recombination events that can produce genomic alterations ranging from deletions and duplications to large-scale chromosomal rearrangements [35]; and (3) through the effects exerted by HERVs and their LTRs that naturally present promoters, enhancers, polyadenylation signals and splice donor sites [5, 36–38] and can regulate also human genes expression in a tissue specific manner [39–49].

In this context, the current HERV-W expression studies seem to be not exhaustive to understand the real effects that these elements can exert. In fact, on the one side, due to their multi-copy nature, it is not always clear from which genomic locus a HERV-W mRNA is transcribed, and, on the other side, the potential effects of such sequences is not solely connected to their expression capacity, but depends also on their localization and their ability to (dys)regulate host functions also through alternative mechanisms behind the presence of a RNA/protein products.

In the light of this, the definition of a precise and updated HERV-W genomic map is a pressing need to better evaluate their role in human health and their real influence on host genome. Here we report a comprehensive analysis of HERV-W sequences presence and distribution within human genome, with a detailed description of the different structural and phylogenetic aspects characterizing the group.

## Results

### HERV-W identification and general classification

In a recent work aimed to the global classification of HERV clades and sequences in the human genome, we reported the presence of 126 elements belonging to HERV-W group [5]. These data were obtained through the bioinformatics tool RetroTector (ReTe), a program package implemented for the identification of ERV full integrations in vertebrate genomes and the attempted reconstruction of the relative ORFs and proteins [50]. For HERV sequences recognition ReTe uses a collection of generic, conserved motifs, a few within *env* and *gag* genes, that can be mutated or lost in defective proviruses [5]. Such “bias” was reported as responsible for the low representation of HERV Class III proviruses that have an aberrant *gag* and may not have an *env* [5]. In the light of this, willing to build an updated dataset of HERV-W sequences in the human genome GRCh37/hg19 assembly, we used a combined strategy based on (1) the ReTe analysis and (2) a traditional Genome Browser BLAT search [51], using the assembled RepBase reference LTR17-HERV17-LTR17 [52] as a query. This integrated approach led to the characterization of a total of 213 HERV-W related sequences: the 126 previously identified by ReTe and 87 additional elements retrieved by Genome Browser BLAT. Indeed, a high proportion of newly identified HERV-W sequences showed huge and recurrent deletions that caused loss of extended portions in *gag*, *pol* and *env* genes (described more in detail in the structural characterization section). Hence, the defective nature of the great majority of HERV-W sequences could be responsible for the underrepresentation of the ReTe outcome, confirming the importance of a double approach in HERV identification.

The main characteristics of HERV-W elements are summarized in supplementary material (Additional file 1: Table S1). We named the HERV-W elements according to their genomic localization, in order to have a unique and direct identification of each sequence. In the presence of multiple sequences in the same locus, the order within the band is expressed with a letter following the alphabetical order as previously described [53]. HERV-W elements occurred on all chromosomes showing no recognizable cluster distribution, except chromosome 16 that apparently do not contain HERV-W proviruses or pseudogenes.

The 213 HERV-W sequences were firstly divided into three categories due to previously reported structural characteristics that mostly address the LTRs portion and that reflect their mechanism of formation [8]: proviruses (65), processed pseudogenes (135) and undefined elements (13). Briefly, with respect to the LTR17 RepBase consensus (780 nucleotides), proviral sequences show complete LTRs (referred here as proviral LTRs) and have been inserted into human DNA by a traditional process of retroviral integration. Proviral LTRs show a traditional composition with two unique regions (U3 and U5) separated by a repeated portion (R), giving a U3-R-U5 structure. As described by Pavlícek et al. [8], pseudogenes are LINE-1 processed HERV-W sequences presenting (1) truncated LTRs (referred here as pseudogenic LTRs), with the 5′ LTR showing a R-U5 structure (start from nucleotide 256 of the consensus) and the 3′ LTR showing a U3-R structure (end at position 326 of the consensus), (2) a poly(A) tail of variable length, and (3) a common TT/AAAA insertion motif and a variable-length (5–15 bp) target site duplication [8]. Finally, undefined elements are sequences that have lost those regions in both LTRs and so remained undefined due to the absence of the signatures described above.

It is interesting to note that our results differed from previous analysis performed a number of years ago on not exhaustive draft versions of the human genome [8, 30, 31] and with the use of different detecting methodologies, leading to discordant results that are not always easy to retrieve and correlate with current data. In fact, on one side, two studies on HERV-W distribution and composition [30, 31] reported a lower number of elements with respect to our dataset. In particular, Voisset et al. described the presence of 70, 100, and 30 HERV-W-related *gag*, *pro*, and *env* regions, respectively, without further indications about their origin [30], while Costas identified a total of 140 HERV-W elements, 73 less than the present analysis. On the other side, when compared to our dataset, the study by Pavlicek et al. reported a higher number of HERV-W sequences (311) [8]. The lack of available supplementary information of Pavlicek HERV-W dataset (e.g. nucleotide sequences or genomic localization) did not allow us to perform a direct comparison with our results. However, Pavlicek et al. HERV-W sequences were retrieved from a draft version of the human genome using the RepeatMasker program that, in the presence of the recurrent and huge deletions such as the ones observed in the HERV-W sequences, could not easily identify the whole elements. Hence, more fragments previously reported as independent elements possibly belonged to the same provirus/pseudogene. This hypothesis seems to be confirmed by a subsequent study where the same dataset has been used for the HERV-W processed pseudogenes length distribution analysis [54]. Such report showed that the most represented length class in Pavlicek dataset enclosed very short sequences (0–0.5 Kb), with a low proportion of >3.5 Kb elements. Differently, in our dataset >90 % of sequences are in the 1–7.5 Kb range, with around 25 % >6.5 Kb. Overall, the use of the Rete software, that relates retroviral elements reconstructing the original chain [50], together with a visual inspection of all aligned sequences plus their flanking sites of integration with respect to the group reference, probably led to more reliable sequence recognition. Furthermore, the overestimation of HERV-W members in Pavlicek dataset could also be due to the possible inclusion of HERV9 sequences, highly related to HERV-W but constituting a separate phylogenetic group [5]. In fact, to avoid such bias we initially included a HERV9 consensus in every HERV-W phylogenetic trees, assuring that none of the sequences classified as HERV-W clustered with HERV9 group (data not shown). Importantly, a significant contribution on the HERV-W group presence in the human genome was recently provided in a study in which the cDNA obtained from HERV-W RNA transcripts in MS patients and controls brain samples was amplified in the *env* region and assigned to single HERV-W loci by Genome Browser BLAT on the NCBI36/hg18 assembly (March 2006) [20]. While the purpose of that study was not a HERV-W group genomic characterization and was biased for *env* sequences analysis, yet it provided a remarkable genomic map of 176 HERV-W loci, enclosing 35 proviruses, in their supplementary material [20]. Noteworthy, with respect to this study, our analysis led to the identification of 37 further HERV-W elements (9 proviruses, 18 processed pseudogenes and 10 undefined sequences), and to a more defined classification of proviruses and processed pseudogenes.

### Structural characterization

In order to characterize the HERV-W structure we firstly aligned and analyzed the 213 sequences dataset with respect to the assembled reference LTR17-HERV17-LTR17, built from RepBase Update consensus sequences for HERV-W LTRs and internal portion [52]. HERV-W sequences showed a typical proviral structure, with the *gag*, *pro*, *pol* and *env* genes flanked by two LTRs. Briefly, the *gag* gene (nucleotides 2718–4191) encodes the structural components of matrix (MA), capsid (CA) and nucleocapsid (NC); the *pro-pol* genes (4195–7692) determine the production of the three viral enzymes Protease, Reverse Transcriptase (RT) and Integrase (IN); and the *env* gene (7720–9348) is responsible for encoding the envelope surface (SU) and transmembrane (TM) elements. The 5′- and 3′ LTRs (1–780 and 9406–10186, respectively) are formed during the retrotranscription process and are identical at time of integration. In addition, almost all HERV-W identified sequences present a 2 Kb long non-coding region, located between 5′ LTR and *gag* gene and characterized by an AG rich expansion of variable length. This portion was previously reported for three cDNA HERV-W clones [11], but neither function or origin has been proposed or demonstrated yet.

**Fig. 1:**
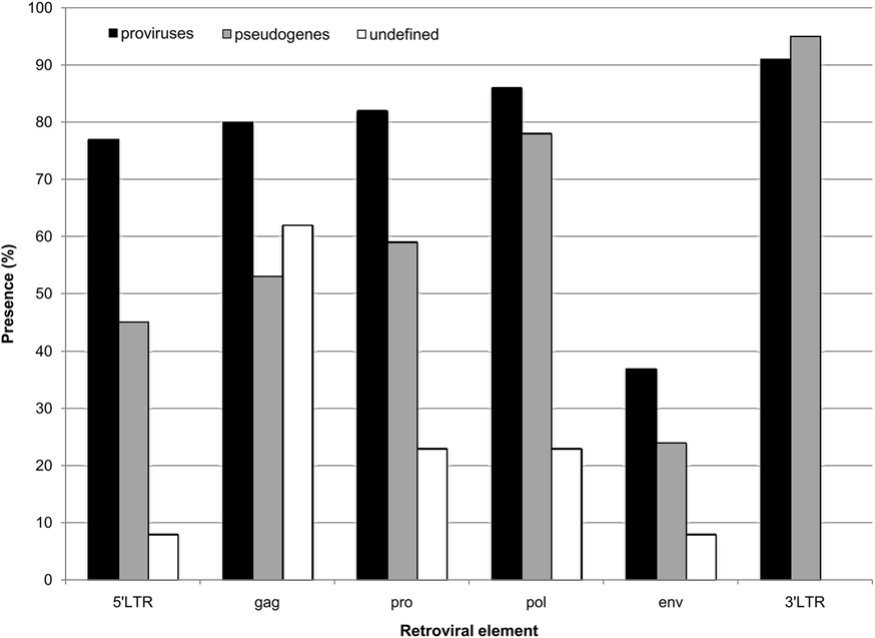
Overview on the HERV-W structural completeness. The presence of each retroviral element in the three classes of HERV-W sequences is depicted. An element is considered as retained if at least the 20 % of the sequence is present (lengths are referred to LTR17-HERV17 reference elements). With respect to the LTR17 RepBase consensus (780 nucleotides), proviral sequences show complete LTRs, while processed pseudogenes present typically truncated LTRs due to the L1-mediated retroposition, lacking U3 in 5′ LTR (R-U5 structure, nucleotides 256–780) and U5 in 3′ LTR (U3-R structure, nucleotides 1–326)

Firstly, comparing proviral versus pseudogenic sequences we asked whether, beside the shorter pseudogenic LTRs, the internal sequence completeness was comparable. We evaluated the presence of each retroviral element (5′ LTR, *gag*, *pro*, *pol*, *env* and 3′ LTR) in the three classes of HERV-W sequences (proviruses, processed pseudogenes and undefined), considering as retained all elements with at least the 20 % of nucleotides with respect to the correspondent element in LTR17 and HERV17 RepBase consensus (Fig. 1). Analysis of all identified HERV-Ws showed that proviral sequences appear to be the most complete, with a better general maintenance of the considered retroviral structures, while pseudogenes, interestingly, frequently lack the 5′ LTR, absent in >50 % of elements, and *gag* and *pro* genes (Fig. 1). Importantly, for all classes, *env* is the most frequently lost element, due to recurrent extended deletions that involve >80 % of the gene. In addition, besides the lack of both LTRs, undefined sequences showed a higher frequency of gene loss, especially in the *pro-pol-env* portions, indicating that deletion processes were not limited to the LTR sequences.

**Fig. 2:**
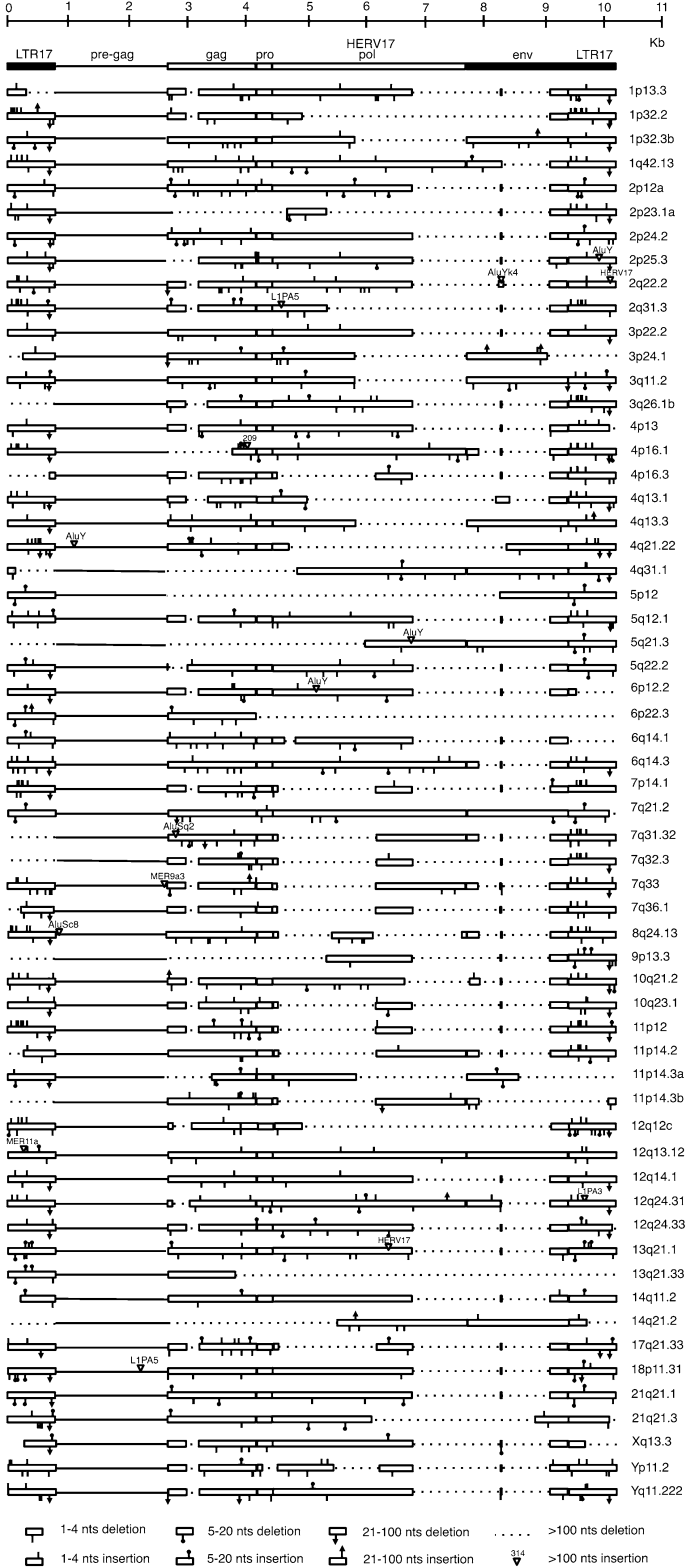
Insertion and deletions of the 59 proviral sequences >2.5 Kb with respect to LTR17-HERV17-LTR17 reference

Secondly, in order to define the group structural characteristics, the 213 HERV-W elements were further analyzed in great detail by annotating all insertions/deletions with respect to the consensus LTR17-HERV17-LTR17, as schematically represented for the 59 proviruses with minimum length of 2.5 Kb in Fig. 2. In comparison to the consensus, in all types of sequences some recurrent deletions clearly affect viral genes, with the loss of some big viral portions: (1) nucleotides 2780–3209 in *gag* gene (45 % of the sequences), (2) nucleotides 4513–6184 and 6797–7692 (IN portion) in *pol* gene (28 and 84 % of the sequences, respectively), and (3) nucleotides 7928–9114 in the *env* gene (85 % of the sequences), with the exception of a small region of about 30 nucleotides at position 8289–8318 that is frequently present despite the flanking deletions. Interestingly, the recurrent loss of *pol* and *env* genes, deleted in the C-terminal IN portion and retaining only the TM intracytoplasmic tail, respectively, possibly suggests a selective removal of regions that were no longer needed in the absence of an active infective transmission.

**Fig. 3:**
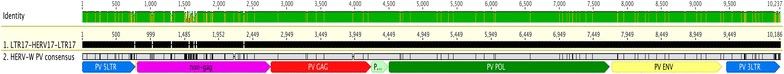
Comparison between HERV-W RepBase consensus LTR17-HERV17-LTR17 (*black*) and the proviral dataset generated consensus (*grey*). Nucleotide identity between the two consensus sequences is represented by the *colored upper bar* (*green* 100 % identity; *greeny-brown* between 100 and 30 % identity; *red* identity <30 %), while single nucleotide differences of the new consensus with respect to LTR17-HERV17-LTR17 are represented with *black lines*. The retroviral LTRs and genes localization is shown below

In addition to these major mutations, the analyses highlighted a greater amount of minor insertions/deletions and single nucleotides substitutions that, overall, allow to specifically identify the uniqueness of each HERW-W sequence. The majority of these variations appear to be randomly distributed among the sequences, as expected from the normal random genomic substitution rate, while a number of them are shared by the great majority of the sequences and characterize their structure with respect to the reference. This analysis allowed also to better defining a new HERV-W consensus generated from our proviral dataset that we graphically compared with the LTR17-HERV17-LTR17 consensus (Fig. 3). Interestingly, the LTR structures of the new HERV-W consensus showed recurrent mutations defining two subgroups of sequences that were used, in combination with the phylogenetic analysis, as key positions for subgroup definition.

### Phylogenetic analysis and HERV-W proposed subgroup classification

**Fig. 4:**
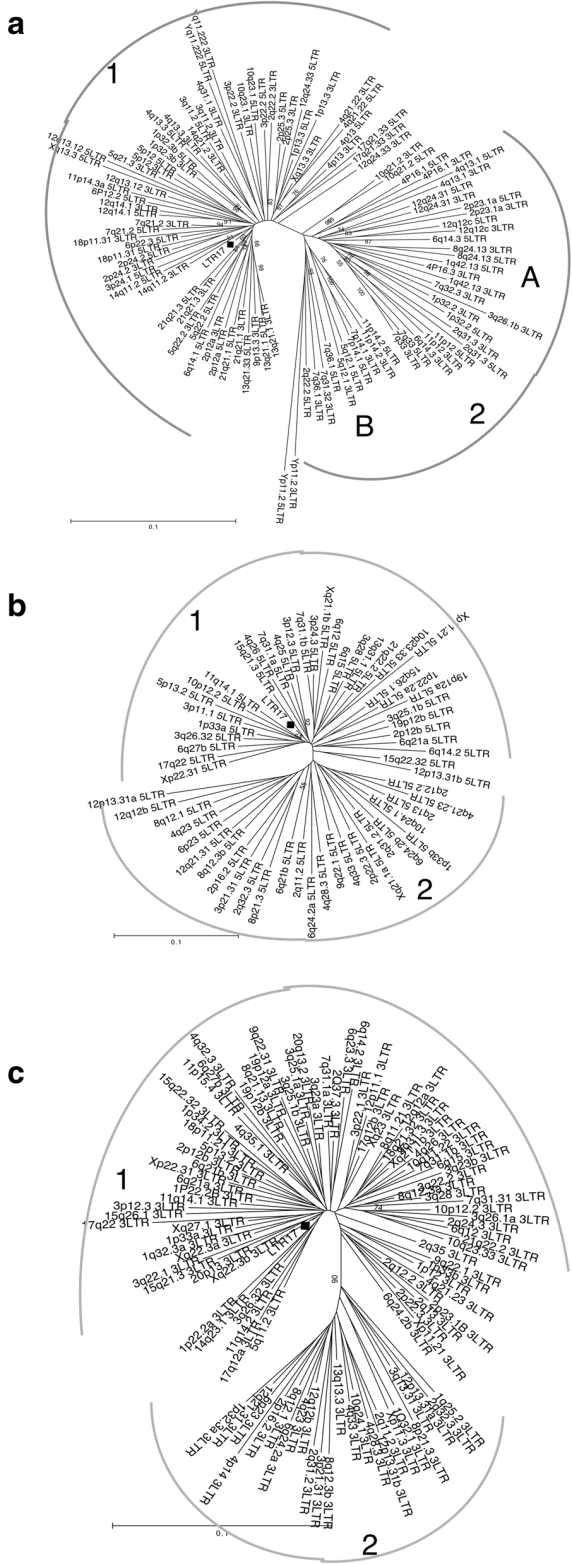
Neighbor joining trees of HERV-W proviruses 5′- and 3′ LTRs (**a**), processed pseudogenes 5′ LTRs (**b**) and processed pseudogenes 3′ LTRs (**c**). RepBase LTR17 consensus is labeled with a *black square*

In order to clarify the phylogenetic and evolutionary relationship within the group, LTRs and viral genes were analyzed through the construction of phylogenetic trees using both a neighbor-joining (NJ) method (Fig. 4; Additional file 2: Fig. S1) and a maximum likelihood (ML) analysis (data not shown). Both analysis yielded similar trees. In addition, since HERV9 sequences have been reported to be highly related to HERV-W, to exclude possible misclassifications, a HERV9 generated consensus [5] was initially included in all trees in order to identify any member of this HERV-W related family that could be misinterpreted during the sequence collection. As expected, the HERV9 consensus was clearly separated from the HERV-W sequences, which grouped together showing a 100 % bootstrap value in every tree (data not shown).

In the case of proviral sequences, the 5′- and 3′ LTRs were analyzed together in the same phylogenetic tree (Fig. 4a). On the contrary, the truncated structure of pseudogenic 5′- and 3′ LTRs only yields a short common region (R; about 90 nucleotides) necessitating a separate analysis (Fig. 4b, c). *gag*, *pol* and *env* genes trees are included in supplementary material in Additional file 2: Fig. S1.

#### LTRs

In LTR trees, the distribution of proviral and pseudogenic sequences in two major clusters allowed us to divide them into two distinct subgroups, named 1 and 2. The subgroup of HERV-W single members is reported in Additional file 1: Table S1. Within the 213 HERV-W group members, 69 % of the sequences belong to subgroup 1 (38 proviruses and 108 pseudogenes), while 24 % of them belonged to subgroup 2 (25 proviruses and 27 pseudogenes). The remaining 7 % was constituted of sequences lacking both LTRs and that, subsequently, could not be classified.

Considering that the subgroup division was generally not well supported by bootstrap values, < 50 % except for pseudogenic 3′ LTR (90 %), the identified HERV-W clusters were further analyzed using common features. The members of each subgroup were aligned and compared with respect to LTR17 reference in order to find characteristic features that could confirm and support the robustness of the classification. In general, subgroup 1 elements were not characterized by significant mutations with respect to the reference sequence, probably because LTR17 and HERV17 consensus were built from a few of these elements, such as the 7q21.2 Syncytin-1 locus. A pairwise distance calculation confirmed that the average identity with LTR17 was around 93 %. Contrarily, subgroup 2 elements showed a lower identity with respect to LTR17 (87 %) and in fact the comparison highlighted the presence of recurrent single nucleotide substitutions. The latter were commonly shared with high frequency in this subset of sequences but not in the other subgroup, and were thus characterized as key mutations for the proposed classification (Table 1). Particularly, in proviral LTRs we identified seven positions with characteristic single nucleotide substitutions with respect to LTR17, which are present in 95–100 % of subgroup 2 members and rarely found (0–3.5 %) among subgroup 1 members. Moreover, in proviral LTRs tree we observed that subgroup 2 elements seemed divided in two further branches, indicated as type 2A (n = 16) and 2B (n = 7) (Fig. 2; Table 1). While both shared the recurrent mutation typical of the main subgroup, each one further shows some additional features found in 90–100 % of sequences and rarely present in subgroup 1 elements. These additional mutations were not exclusive of each branch, but were present also in the other subgroup 2 type of elements with frequencies from 19 % up to about 50 % and were thus reported for completeness but not considered for phylogenetic purposes.

**Table 1:** Recurrent mutations in HERV-W subgroup 2 LTRs

Position (nt)^a^	Substitution^b^	Frequency^c^			
		PV^d^ subgroup 2	PG^e^ subgroup 2	Solo LTRs subgroup 2	Subgroup 1
43	C>T	100	100	98	0.7
95	C>T	100	95.8	96	3.4
100	T>C	97.3	100	95	2.2
180	C>T	97.3	100	95	0
254	A>G	97.3	96	87	1.4
706	A>G	97.4	73.3	88	1.7
765	G>A	95	73.3	90	1.7

		Frequency^c^		
Position (nt)	Substitution^b^	PV^d^ subgroup 2A	PV^d^ subgroup 2B	Subgroup 1
*Type 2A additional mutations*				
456	C>T	100	41.7	10.5
498	A>G	92.6	33.3	3.5

Position (nt)^a^	Substitution^b^	PV^d^ subgroup 2B	PV^d^ subgroup 2A	Subgroup 1
*Type 2B additional mutations*				
133	A>G	100	48.1	0
188	C>A	90	19.2	0
252	C>G	90	40.7	1.7

^a^Nucleotide positions are referred to RepBase Update LTR17consensus

^b^Substitutions are indicated with the original nucleotide and the acquired new variant separate by the symbol >

^c^Relative percentage based on the total of sequences that hold the position in an alignment

^d^Proviruses

^e^Pseudogenes

The identified key substitutions were then investigated also in the pseudogenic HERV-W dataset, where their strong relation with the sequences distribution in the NJ trees was confirmed for the first 5 positions (96–100 % frequency in subgroup 2 versus 0–3.5 % in subgroup 1), while the last two mutations were shared among about the 75 % of sequences (Table 1). Due to the pseudogenic LTRs truncated structure, the subgroup division was evident in the 3′ LTRs tree (U3-R, positions 1–326 in LTR17) where 5 key positions out of 7 are maintained. The pseudogenic 5′ LTRs (R-U5, positions 256–780) harbor instead only the two less represented key positions and showed a more confused topology, underlining the importance of the described substitutions in the phylogenetic asset of the group.

#### Extended analysis of HERV-W genomic LTRs

Considering the relevance of LTR structural characteristics for HERV-W classification purposes, we retrieved via Genome Browser BLAT about 800 HERV-W LTRs present in hg19 assembly. This wider dataset has been used to assess the global reliability of the subgroup definition. The NJ tree analysis performed supported our classification, with a tree that resembled the topology observed for proviral and pseudogenic LTRs (Additional file 3: Fig. S2) and showed a comparable distribution of solitary elements between the two subgroups (71 % classified as subgroup 1 and 29 % as subgroup 2). When investigated for recurrent substitutions, the key positions defined for subgroup 2 were confirmed as commonly shared in 87–98 % of the subgroup members and rarely present (1–6 %) in the rest of the whole HERV-W LTRs dataset.

#### Retroviral genes

The NJ trees built for the retroviral *gag*, *pro*, *pol* and *env* genes did not highlight the presence of any subgroup (Additional file 2: Fig. S1), and the nucleotide analysis confirmed that the sequences share a comparable grade of homology. This result demonstrated that the phylogenetic relevant variations within the HERV-W group are located in the LTR elements.

A LTR-based classification was previously suggested by Costas, that identified three distinct HERV-W subfamilies named 1, 2 and 3, on the basis of nucleotide differences described in a shorter version of the 3′ LTR, with a truncation in correspondence to position 326 of LTR17, typical of pseudogenes [31]. Our data indicate instead that the HERV-W main subgroups are only two: subgroup 1 (associated to Costas subfamily 3) and subgroup 2 (related to Costas subfamilies 1 and 2). Subgroup 2 key mutations enclose the 5 mutations observed by Costas plus 2 more in the 3′ LTR terminal portion. With respect to the previous classification, the one we propose is primarily based on a phylogenetic analysis, corroborated by the presence of high frequency key positions found in both 5′ and 3′ full-length LTRs and confirmed for the first time in a comprehensive HERV-W solitary LTRs dataset.

### Time of integration

It is known that, at time of integration, the 5′- and 3′ LTRs of the same provirus are identical [55] and accumulate random substitution in an independent way. Hence, to assess the HERV-W group estimated age we assumed for the human genome a substitution rate of 0.13 %/nucleotides/million year [56] and used this rate to assess the action of divergence on each HERV-W sequence. Based on this assumption, we calculated the percentage of divergent nucleotides (D %) (1) between the 5′- and 3′ LTRs of each HERV provirus; (2) between each LTR (proviral and pseudogenic) and a generated consensus for each subgroup and (3) between a 150–300 nucleotides region of each HERV-W internal element *gag*, *pro*, *pol* RT, *pol* IN and *env* genes (proviral and pseudogenic) and a generated consensus. Regarding the two consensus-based approaches, in consideration that the substitution rate acts randomly on each sequence, the subgroup-generated consensus should ideally represent the ancestral situation.

**Fig. 5:**
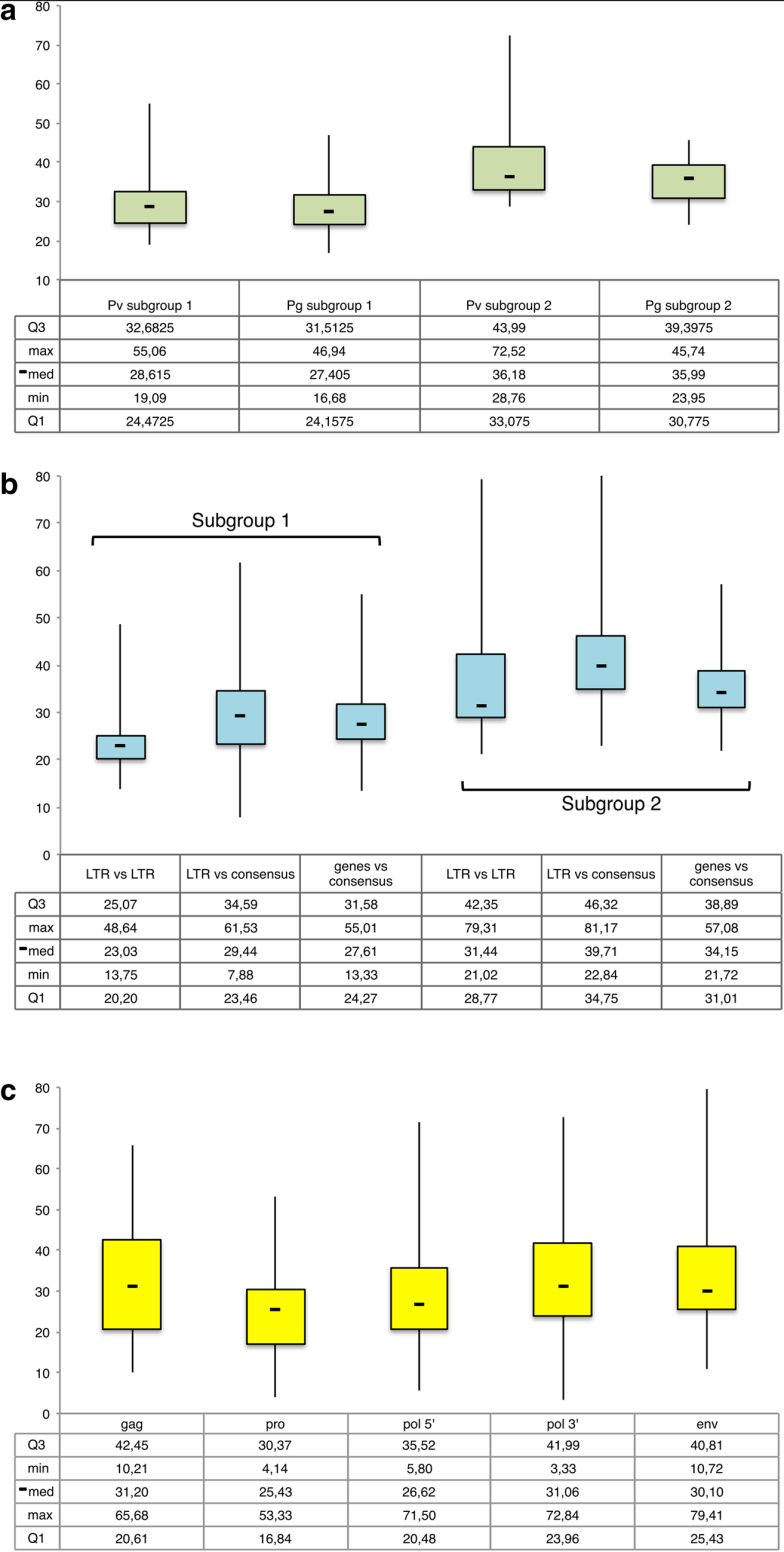
Boxplot representations of HERV-W subgroups divergence based estimated period of integration. The approximated age (in million years) was calculated considering the divergence values between the 5′- and 3′ LTRs of the same provirus (only for proviral sequences); between each LTR and a generated consensus for each subgroup and between a 150–300 nucleotides region of each HERV-W internal element *gag*, *pro*, *pol* RT, *pol* IN and *env* genes and a generated consensus (proviruses and pseudogenes). **a** Averaged values of age obtained for each sequence, after the sequences division in proviruses and pseudogenes for each subgroup. **b** Single method estimations for the two HERV-W subgroups. **c** Highlight of the heterogeneous action of the divergence at different genic regions

The obtained divergence values were used to calculate the age of the HERV-W sequences. For all three approaches the calculation is based on the relation T = D %/0.13 %, where T is the estimated time of integration (in million years) and 0.13 % is the applied genomic substitution rate per million year. For the divergence between 5′- and 3′ LTR of the same sequence, the obtained T value was divided by a factor of 2, considering that each LTR evolved and accumulated mutations independently. The reported time of integration (Additional file 1: Table S1) has been calculated as the average resulted from the methods used (Fig. 5). In particular, the estimated time of integration of proviral and pseudogenic sequences for both subgroups 1 and 2 (Fig. 5a) describes for the first time the HERV-W dynamic of insertion into the human genome, suggesting that: (1) the first HERV-W integrations involved subgroup 2 and occurred more than 40 million years ago, with a diffusion of proviral and pseudogenic sequences until about 30 million years ago; (2) HERV-W subgroup 1 sequences are significantly younger with respect to subgroup 2 members (p < 0.0005), and have been acquired mostly between 35 and 25 million years ago, occurring in average about 8 million years later than subgroup 2; (3) it is interesting to note that, for both subgroups, the dissemination of proviruses and processed pseudogenes took place virtually simultaneously. Moreover, despite both subgroups proviruses were processed by the LINE machinery to generate processed pseudogenes, the mechanism was more frequent for subgroup 1 proviruses (1:2.5 ratio with the number of related pseudogenes) than for subgroup 2 integrated elements (1:1 ratio). The reason for this is at the moment unclear. We attempted to connect the single pseudogenic sequences to the original generating proviruses by a phylogenetic analysis of LTRs and major genes, expecting that the pseudogene elements could cluster with their respective HERV-W locus of origin. However, the great majority of pseudogenes clustered with different proviral loci according to the sequence portion considered (data not shown). Hence, this result, together with the estimated time of diffusion of pseudogenic elements, suggests that the HERV-W processed pseudogenes have acquired a comparable amount of heterogeneity since their mobilization by LINE elements, and it is thus not possible to univocally assign each one to a single proviral locus.

It is important to note that the traditional sole comparison of the two LTRs of the same sequence would not be sufficient for a reliable estimation. In fact: (1) the LTR versus LTR method could not be applied at all to pseudogenic sequences, representing the 63 % of the whole dataset, due to the short region in common between the 5′- and 3′ LTRs; (2) also in the case of proviral sequences, the lack of one or both LTRs make possible such calculation only for the 70 % of proviral sequences (23 % of the total HERV-W members). The two additional approaches completed and improved the time of integration estimation, allowing to consider a larger subset of elements (94 % of the total HERV-W members) and to represent also pseudogenes and older and less intact sequences, which were not previously taken into account. Importantly, the combination of multiple divergence calculations provided significant improvements also in age estimation reliability and precision. The expression of each HERV-W sequence time of integration through the use of an averaged value allowed to determine the standard deviation and to reduce estimation biases related to outliers and different selective pressure that are reported to interest LTR elements with respect to the rest of the retroviral genome [38] (Fig. 5b). Data showed that some proviruses had a 0.3–2 folds higher age estimation when calculated using the LTR versus consensus method as compared with the LTR versus LTR method. Despite the absence of a clear explanation, it is possible to speculate that the exogenous viruses that gave rise to these sequences harbored some nucleotide differences in their LTRs that are not properly represented in the consensus sequence, built on the majority of viruses, leading to an apparent higher amount of mutations. In addition, data showed a higher divergence in the *gag*, *pol*-3′ (including IN) and *env* portions, leading to a older age estimation with respect to the internal *pro* and *pol*-5′ regions (Fig. 5c), thus suggesting different mutation rates according to the specific viral portions.

Taken together, these results suggest that the HERV-W group integration started about 40 million years ago at the time of the Catarrhini primates, after the divergence between New World Monkeys and Old World Monkeys. This is in line with previous studies [31, 57], which were based on the presence of HERV-W PCR products in different Old World Monkey blood samples [57], or on the divergence calculation among HERV-W subfamilies [31], and gave thus just a general overview of primates HERV-W group acquisition. In the present study, the time of insertion has been estimated for each single HERV-W locus through at least two different methods of age calculation, providing a precise and exhaustive picture of the group diffusion among primates, with a rather long period of activity that took place until 25–20 million years ago.

The estimated age of the single HERV-W sequences was generally also supported by the identification of each locus orthologous in primates until the Oldest Common Ancestor (O.C.A.) (Additional file 1: Table S1). Results showed that the great majority of sequences are shared from human to Rhesus Macaque (61 %) or to Gibbon (31 %), with an entry that must be occurred after their separation from the Platyrrhini parvorder (40 million years ago) and before their divergence from the evolutionarily younger hominoids, occurred around 30 (Rhesus Macaque) and 20 (Gibbon) million years ago [58]. Few elements were also found starting only since Orangutan (12), Gorilla (3) and Chimpanzee (2) (Additional file 1: Table S1), but in these cases the estimated age was higher than expected. This probably suggests that such sequences were lost in older primates, even though their absence in Rhesus and Gibbon could be also due to a lower efficiency of Genome Browser comparison between the human genome and the most ancient Catarrhini assemblies. Finally, a single HERV-W element was found only in the human genome assembly hg19, on locus 12q13.3. This data is unexpected because no human specific HERV-W elements have been reported so far, but could not be supported by reliable age estimation due to the shortness of the sequence (about 1500 nucleotides) and the lack of both LTRs.

### PBS type and gammaretroviral features

**Fig. 6:**
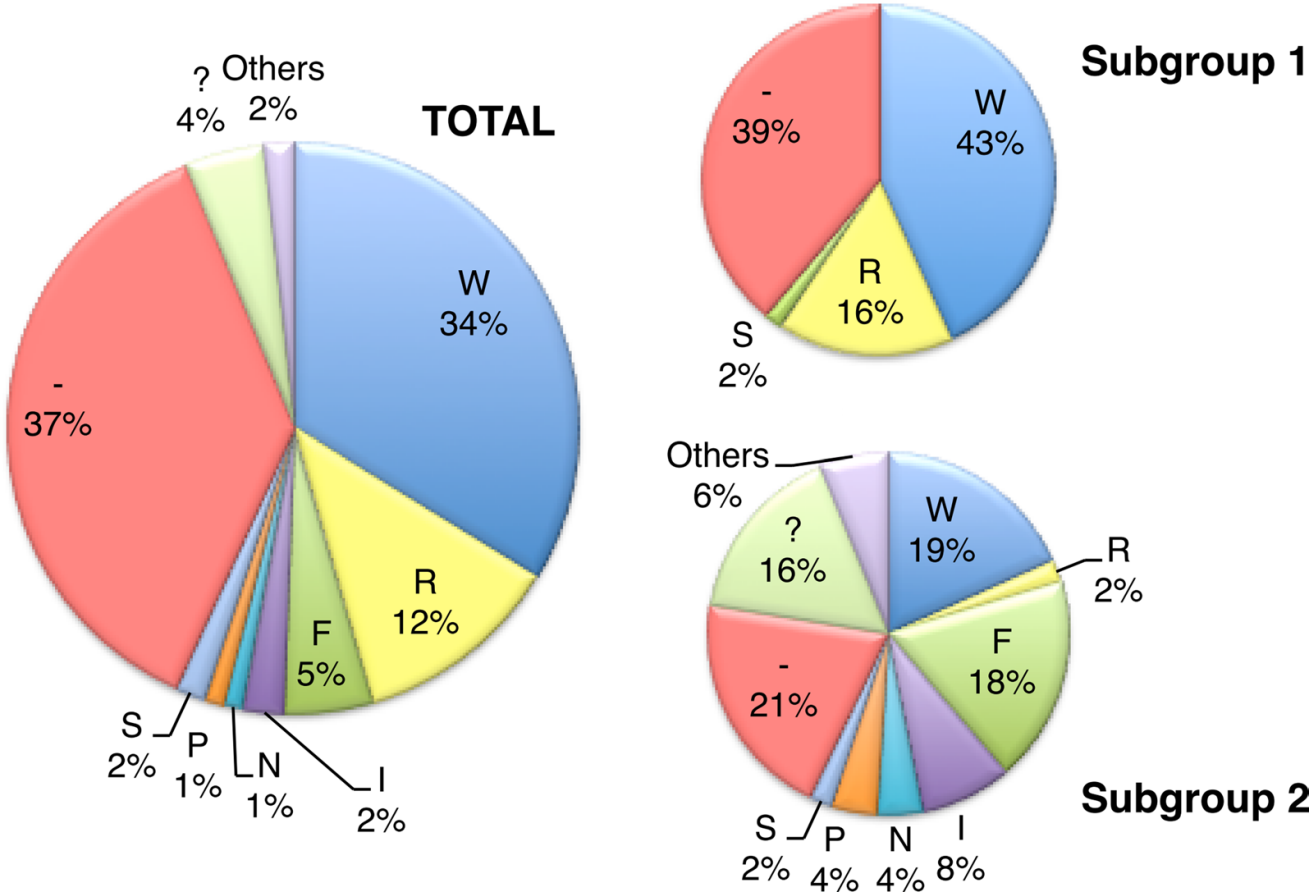
PBS types among all HERV-W sequences and diversity between subgroup 1 and subgroup 2. The PBS types are identified by the amino acid single letter of the corresponding cellular tRNA. W = tryptophan, R = arginine, F = phenylalanine, I = isoleucine, S = serine, P = proline. “Others” category encloses Leucine (L), Glutamic Acid (E) and Glycine (G), each found only in one sequence. Elements that lost the PBS sequence (–) or with PBS with ambiguous assignation (?) are also included

**Fig. 7:**
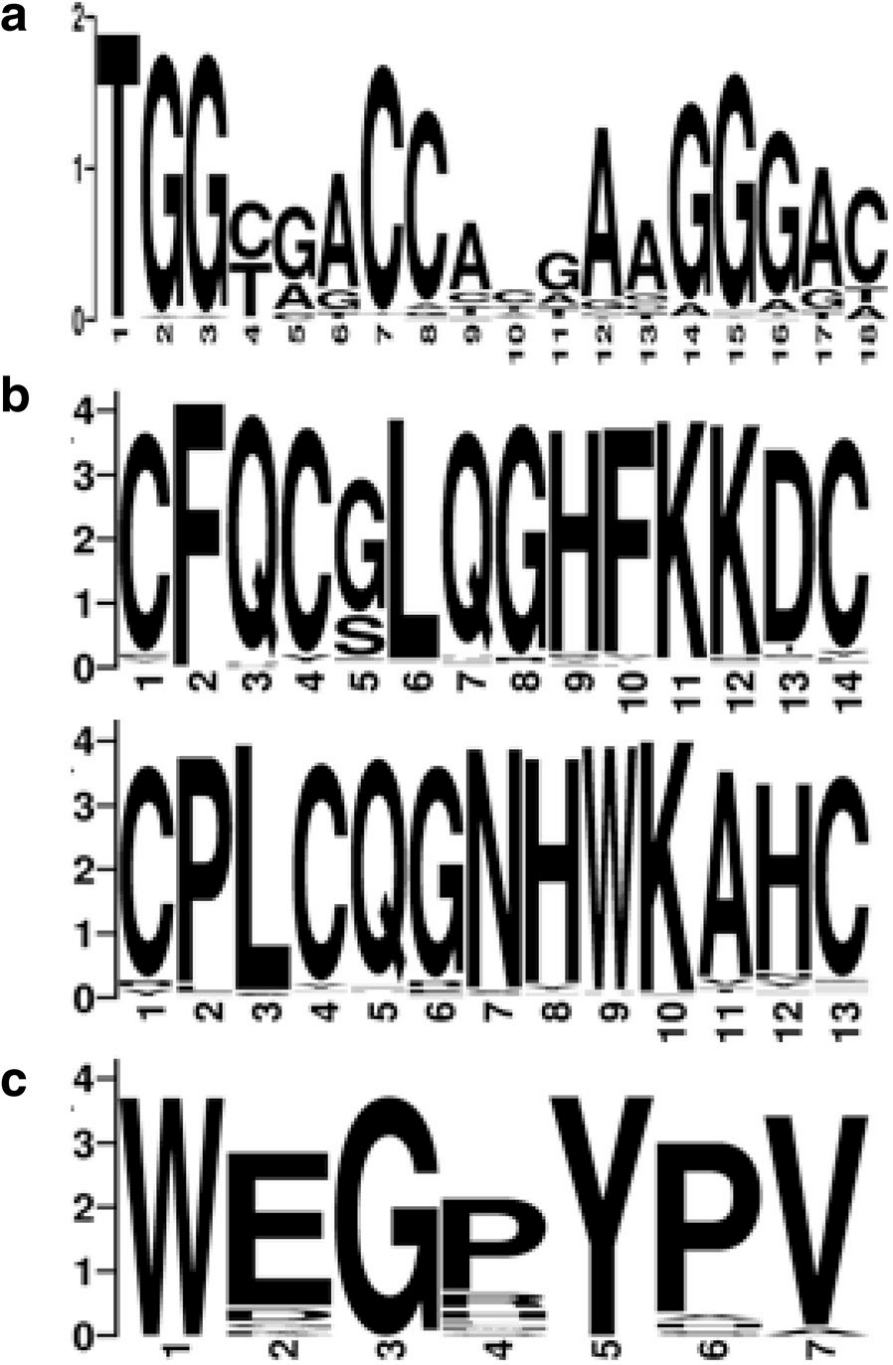
Logos representing the HERV-W main features. **a** PBS nucleotide sequence; **b** Gag nucleocapsid Zinc fingers amino acid composition and **c** Pol IN C-terminal GPY/F motif amino acid composition. The overall height indicates the sequence conservation, while the height of symbols indicates the relative frequency of each amino or nucleic acid. Created at http://weblogo.berkeley.edu/logo.cgi

The PBS type has been historically used to identify the different HERV groups that were commonly designated with the amino acid single letter of the corresponding tRNA. Currently this nomenclature is not considered a sufficient and reliable taxonomic marker, especially because it is not based on HERV phylogeny [5, 59]. In the analyzed HERV-W elements, the PBS was present in 111 sequences and was located approximately 4 nucleotides downstream the 5′ LTR (from nucleotide 4 to 21 in HERV17 consensus). The PBS type of the single sequences is reported in Additional file 1: Table S1, while a graphical overview of the PBS types found in the entire HERV-W dataset and in each subgroup is provided in Fig. 6. In general, Tryptophan (W) was as expected the most common PBS type: it was found in a total of 60 sequences, representing about the 58 % of the identified HERV-W PBSes. Therefore, noteworthy, about half of HERV-W elements analyzed had a non-W PBS type, confirming the relatively low taxonomic value of this feature. Particularly, Arginine PBS was rather common (R, 21), followed by Phenylalanine (F, 9), Isoleucine (I, 4), Serine (S, 3) and Proline (P, 2). Other PBS types found in single HERV-W sequences were Leucine (L), Asparagine (N), Glutamic Acid (E) and Glycine (G). In the remaining eight elements, the PBS sequence was present but it was not possible to unambiguously assign it to a single tRNA. Interestingly, subgroup 1 elements retaining the PBS sequence showed a more homogeneous situation, presenting almost the 100 % of W or R as putative tRNA usage. This was expected since the W codon is the most commonly associated to the HERV-W group, and the R one differs from it only slightly, and sometimes the two codons may overlap due to a single nucleotide shift in the PBS sequence [5]. Differently, subgroup 2 elements revealed a more heterogeneous PBS type usage, including all the unusual tRNA sequences and all the ambiguous PBSes with no clear assignment. These atypical PBSes are probably derived from the accumulation of several substitutions, in accordance with the older appearance and the longer permanence of the sequences in primates genome. To summarize the general variation of the PBS sequence within HERV-W group we generated a logo (Fig. 7a) in which the letter height is proportional to the nucleotides conservation at each position. As expected, the TGG starting nucleotides, which are shared by almost all the PBS types, were the most conserved among the 18 bases analyzed. Interestingly the middle portion of the sequence showed a high variability, especially at positions 4–6 and 11, indicating a rather large diversity of PBSes in HERV-W group.

We have also identified and analyzed structural features typically shared among retroviral sequences within the same genus, that can be used as taxonomic and phylogenetic markers [7]. As previously reported [7], the main gammaretroviral features are (1) one nucleocapsid Zinc finger motif, involved in the retroviral RNA interaction during packaging [60]; (2) the C-terminal polymerase IN GPY/F motif, that binds the host DNA and could have a role in the integration specificity [61, 62] and (3) a nucleotide frequency bias determined by the action of encapsidated host RNA editing systems [7].

The *gag* nucleocapsidic Zinc finger, corresponding to nucleotides 4021–4062 in the RepBase assembled LTR17-HERV17-LTR17 reference sequence, has a typical CX_2_CX_4_HX_4_C amino acid motif. It was found in almost all sequences that retained the harboring genetic region, with a higher prevalence in proviral sequences that were also the most complete in term of genetic composition. Moreover, noteworthy, a second Zinc finger was identified in 96 % of the sequences (nucleotides 4093–4130). This second Zinc finger has a modified structure with respect to the usual one, showing the loss of one of the variable residues (CX_2_CX_3_HX_4_C). The amino acid composition of the two motifs was highly conserved as shown in Fig. 7b. The presence of a second Zinc finger was not previously reported for HERV-W group, and its structure is consistent with the second Zinc finger found in a subset of HERV-H sequences, another gammaretroviral HERV group [63]. However, while for HERV-H a correlation between the presence of this second motif and the age of sequences was proposed, for HERV-W we could not observe such correlation (data not shown).

The IN domain contains a GPY/F motif, a stretch of conserved amino acids with the general WXnGPYXV structure corresponding to nucleotides 7501–7521 in the reference sequence. Considering that the C-terminal part of the polymerase gene was deleted in 85 % of sequences, in the remaining few members the GPY/F feature was found with a 100 % frequency. Also for this feature the logo analysis showed a conserved amino acid sequence (Fig. 7c).

Regarding the nucleotide composition, HERV-W members present a weak bias in purines, tending to be richer in Adenine (about 30 %) and poorer in Guanine (around 22 %) (data not shown). Among Gammaretroviruses an impoverishment of G nucleotide was previously observed for HERV-H group in association with an higher content of Cytosine [63], while the G to A hypermutation condition was reported for HERV-K group [64] and is a well known effect of the APOBEC3 defensive action against HIV-1 Lentivirus [65]. Hence, it is possible to speculate that this editing system could have played a role as a control mechanism to limit HERV-W and other endogenous elements mobility during evolution [66], also considering APOBEC3 ability to greatly inhibit the LINE mediated transposition of other retroelements [67].

### Genomic context of insertion

The current major field of HERVs investigation is their expression and coding capacity, however, the impact of these sequences on the host depends also on their genetic surrounding. The context of integration can, in fact, modulate HERVs physiology, and HERV sequences inserted in proximity of human genes are known to be able to influence their expression [39–41, 43, 45, 48, 49]. As reported for other HERV groups [68], the analysis of the genomic context of all 213 HERV-W confirmed that the majority of sequences are located in intergenic regions, with the exception of 80 elements inserted into human genes.

In particular, 55 elements (26 %) are inserted into human coding genes, mostly exclusively into intronic regions (53/55) (Table 2). These elements showed a strong anti-sense orientation with respect to the surrounding gene (43/55) that was more frequent for pseudogenic sequences than for proviruses (84 and 67 %, respectively). The fact that HERV-W intronic elements are present mostly in antisense orientation could reflect a bias due to an evolutionary and post-insertional selection [69]. Noteworthy, while most of the identified sequences were already characterized for their genomic context [20, 70], 8 of them are reported for the first time to be inserted in human genes. Based on Genome Browser annotations, pseudogene 21q22.2 resulted from an overlap in antisense orientation with the first two exons of IGSF5 gene, that produces a protein involved in junction and adhesion formation, and with a corresponding IGSF5 highly similar mRNA found in placental tissues (AK092516). Interestingly, more than half of the 55 genes that held HERV-W sequences were reported to be positively associated with a disease or a pathologic trait, that in most cases affect the neurological and the cardiovascular systems (Table 2). Considering that the genomic context of integration and the orientation of HERV-W sequences appeared to influence their expression [70], the present mapping of those elements may aid in the understanding the potential effects of these integrations on human health and to direct further investigations to the genes involved.

**Table 2:** HERV-W genomic context: insertions into human coding genes

HERV-W	Human gene	Gene or relative protein function and associations
1p34.2 (+)	*HIVEP3* Int 1(−)	Transcription factor, binds Ig and T-cell receptors recombination signal
1q25.2 (−)	*RASAL2* Int 1 (+)^b^	RAS superfamily of small GTPases protein activator like. Associations: BMI, weight
1q42.13* (−)	*ZNF678* Int 2 (+)^a,b^	Zinc Finger protein. Associations: body height
2p23.1a* (+)	*LCLAT1* Int 2 (+)^b^	Predominantly remodels anionic phospholipids in endoplasmic reticulum
2p16.2 (+)	*ASB3* Int 1/2 (−)^b^	Suppressor of cytokine signaling proteins and their binding partners
2q22.2* (−)	*KYNU* Int 2 (+)^a,b^	NAD cofactors biosynthesis from tryptophan. Associations: body height, cholesterol, schizophrenia
2q24.3 (−)	*COBLL1* Int 2 (−)^a^	Cordon bleu WH2 repeat protein-like 1. Associations: BMI, Cholesterol, HDL, triglycerides, stroke, response to statin therapy, anthropometric sexual dimorphism
2q31.2a (−)	***AGPS*** Int 1 (+)^b^	[603051] Mutations are cause of rhizomelic chondrodysplasia punctata type 3
2q35 (−)	*DIRC3* Int 1 (−)^b^	Disrupted in renal carcinoma long non-coding RNA. Associations: diabetes mellitus
3p22.2* (−)	*SLC22a14* Int 1 (+)^b^	Solute carrier transmembrane protein
3q22.1 (−)	*NEK11* Int 14/13 (+)^b^	Never In mitosis kinase. Involved in DNA replication and G_2_/M checkpoint response to DNA damage. Related to embryonic lethality and preeclampsia
3q23b (+)	*XRN1* Int 1 (−)^b^	Exoribonuclease involved in Long noncoding RNA decapping and miRNA regulation
3q26.32 (+)	*ZMAT3* Int 2/3 (−)^a,b^	Zinc finger matrin. Acts as a bona fide target gene of p53/TP53
4p16.3* (−)	*ZNF595* Int 3 (+)	Zinc finger protein. Function as transcription factor
4p16.1* (+)	*ACOX3* Int 1 (−)^a,b^	Oxidizes the CoA-esters of 2-methyl-branched fatty acids
4q31.3 (+)	***ARFIP1*** Int 2 (+)^b^	ADP ribosylation factor interacting protein1. [605928] Enhance the cholera toxin activity
5q12.1* (+)	*DEPDC1B* Int 2 (−)^b^	Significantly upregulated in nonsmall cell lung carcinoma cell lines (reduced patient survival)
5q22.2* (+)	*ACOT13* Int 1 (+)	Acyl-CoA thioesterase. Involved in regulation of lipid composition and metabolism
6q12 (+)	***EYS*** Int 13 (−)^b^	[612424] In photoreceptor layer: mutated in autosomal recessive retinitis pigmentosa
6q14.3a* (−)	*TBX18* Int 7 (−)^a,b^	Role in embryonic development. Associations: cholesterol, coronary disease
6q21a (+)	*ATG5* Int 6 (−)^a,b^	Autophagy related apoptosis specific protein. Associations: lipoproteins, LES
6q21b° (+)	***PDSS2*** Int 2 (−)^b^	Prenyl (decaprenyl) diphosphate synthase, subunit 2. Synthesizes the side-chain of coenzyme Q. [610564] Coenzyme Q10 deficiency, primary, 3: fatal encephalomyopathy and nephrotic syndrome
6q21c (+)	*SLC16A10* Int 1 (+)^a,b^	Na^(+)^-independent transport of aromatic amino acids across the plasma membrane. Associations: cholesterol, LDL
6q24.2a (−)	*AIG1* Int 1 (+)^b^	Androgen-induced. Associations: C-reactive protein, insulin, myocardial infarction
7p21.1 (−)	*BZW2* Int 3 (+)	Homo sapiens basic leucine zipper and W2 domains 2
7p14.1* (−)	***SUGCT/C7orf10*** Int 1 (+)^b^	[609187] Mutations are associated with glutaric aciduria type III. Others: BMI, fat distribution, cardiomegaly, coronary disease, pancreatic and prostatic neoplasms
7q31.1a (+)	*NRCAM* Int 2 (−)^a^	Neuronal Cell Adhesion Molecule. Associations: autism, obsessive compulsive disorder, schizophrenia
7q31.1b (−)	***FOXP2*** Int 2 (+)^a,b^	[605317] Required for development of speech and language regions of the brain during embryogenesis. Associated to speech-language disorders
8p21.3 (+)	*SLC18A1* Int 10/11 (−)^b^	Involved in vesicular transport of biogenic amines. Associations: bipolar disorder, major depressive disorder
8q12.3a (−)	*NKAIN3* Int 3 (+)	Na+/K+ transporting ATPase interacting proteins. Associations: mental competency, neuroblastoma, stroke
8q12.3b (+)	***CYP7B1*** Int 1 (−)^b^	[603711] Cyp450 enzyme. Associations: bile acid synthesis congenital defect, spastic paraplegia. Others: Alzheimer disease, lipoproteins, schizophrenia
8q21.11 (+)	***UBE2*** ***W*** Int 2(−)^b^	Ubiquitin-conjugating enzyme. Along with ubiquitin-activating (E1) and ligating (E3) enzymes, coordinates the ubiquitin addition to proteins. [614277] Interacts with FANCL and regulates the monoubiquitination of Fanconi anemia protein FANCD2
8q21.13 (+)	*ZNF704* Int 2 (−)^b^	Zinc finger protein
9p24.1 (+)	***PTPRD*** Int 12 (−)^b^	Protein tyrosine phosphatase, receptor type, D. [601598] Restless Legs Syndrome. Associations: asthma, BMI, cholesterol, lipids, lipoproteins, triglycerides, diabetes
9p13.3* (−)	*CD72* Int 1 (−)^a,b^	B-cell proliferation and differentiation antigen. Associations: lupus erythematosus
10q23.33 (−)	***CYP2C19*** Int 6 (+)^b^	[124020] Cyp450 enzyme, responsible for therapeutic agents metabolism. Associated to metabolic defects and variants
10q24.1 (−)	***ENTPD1*** Int 1 (+)^b^	[601752] Triphosphate Diphosphohydrolase. Associated with Spastic Paraplegia
11p14.2* (−)	***ANO3*** Int 14 (+)^a,b^	[610110] May act as a chloride channel. Associations: Dystonia 24. Others: bmi, obesity, c-reactive protein, cholesterol, coronary disease, schizophrenia
11q14.1 (−)	*AAMDC* Int 2 (+)^b^	Adipogenesis associated Mth938 domain containing
11q14.2 (−)	*PRSS23* Int 2 (+)^b^	Encodes a conserved member of the trypsin family of serine proteases
12p13.31b (−)	*RIMKLB* Int 5 (+)^b^	Catalyses ATP-dependent condensation of NAA and glutamate to produce NAAG
12q23.3 (+)	*SLC41A2* Int 1 (−)	Solute carrier family 41member 2
13q13.3 (+)	*ALG5* Int 7/8 (−)^b^	Participates in N-linked glycosylation of proteins
14q11.2* (+)	*TCRA* Int 1 (+)^b^	T cell receptor alpha locus
14q21.2* (−)	*FAM179B* Int 7 (+)	Homo sapiens family with sequence similarity 179 member B
14q23.1 (+)	*C14orf37* Int 4 (−)^b^	Associations: attention deficit disorder with hyperactivity
17q12a (+)	*SLFN14* Int 3 (−)^b^	Implicated in regulation of cell growth and T-cell development (studies in mouse
17q12b° (−)	*ACACA* Int 2/6 (−)^b^	Biogenesis of long-chain fatty acid. Associations: BMI, breast cancer
17q22 (−)	*STXBP4* Int 8 *(+)* ^b^	Translocation of transport vesicles from cytoplasm to plasma membrane, like the insulin-stimulated GLUT4 translocation in adipocytes. Associations: BMI, cholesterol
19p12a (+)	***ZNF90*** Int 1 (+)^b^	Zinc finger protein 90. May be involved in transcriptional regulation. [603973]
19q13.2a (+)	*ZNF780A* Ex 9 (−)^b^	Zinc finger protein 780A
19q13.2b (−)	*CYP2A7* in 1 (−)^b^	Cytochrome P450, family 2, subfamily A, polypeptide 7
21q22.2 (−)	*IGSF5* Ex 1–2, Int 1 *(+)* ^b^	Participates at tight-junctions (kidney, gut) or acts as adhesion molecule (testis). Associations: coronary disease, lipoproteins, Parkinson disease, stroke
Xp11.21 (−)	*FAAH2* Int 7 (+)^b^	Degradation and inactivation of bioactive fatty acid amides
Yq11.222* (+)	*CD24* Int 1 (−)	Mature granulocytes and B cells surface antigen

Proviruses and undefined sequences are labeled respectively with * and °. For HERV-W sequences and genes, the strand direction is reported into round brackets. Bold genes are listed as OMIM diseases associated and the relative accession number is reported into square brackets. Underlined genes are reported to be positive associated with specific phenotypes in UCSC Gene annotations

^a^Already reported in Li et al. [70]

^b^Already reported in Schmitt et al. [20]

In addition, 25 HERV-W loci were integrated into 29 human non-coding genes (Table 3) of which the great majority (22) are reported here for the first time. These elements were mostly inserted into regions associated with the production of long non-coding RNA (lincRNA) and microRNA (miRNA), regulatory molecules that operate on different levels of gene expression. These HERV-W proviruses (6), pseudogenes (17) and undefined sequences (2) showed different characteristics with respect to the loci observed into coding regions. Firstly, despite even in this case a majority of intron localization (26) was observed, 8 of them were co-localized with 9 exons, frequently situated at the transcriptional start site of the non-coding gene. Secondly, in this case the antisense bias was not present, since 19 out of the 29 non-coding genes showed the same orientation with respect to the HERV-W inserted elements. These observations suggest that the LTRs of these HERV-W could provide regulatory signals for lincRNA, as already highlighted for HERV LTRs in general [71]. The great majority of non-coding genes are still uncharacterized, but some elements were reported to be associated with post-transcriptional regulation (MIR5684) or related to proteins involved in lipid transfer and proteolytic activity (STARD7-AS1 and PRSS23). Overall, the percentage of HERV-W sequences inserted into human genes (36 %) is higher than the percentage of bases spanned by human genes (24 %) [1, 72] suggesting that integration events could have been biased for genic or against intergenic regions.

**Table 3:** HERV-W genomic context: insertions into human non-coding genes

HERV-W	Human gene	Gene function and associations
1p12 (−)	*LOC101929147* Int 4 (+)	Uncharacterized antisense long non-coding RNA
1p13.3* (−)	*TCONS_00000271* Int 3 (+)	Large intergenic non coding RNA
1q32.1 (−)	*LOC284581* Int 1 (+)^b^	Uncharacterized antisense long non-coding RNA
2q11.2 (−)	*STARD7-AS1* Int1 (+)^b^	StAR-related lipid transfer domain protein 7 antisense long non coding RNA (LOC285033)
2q24.3 (−)	*TCONS_00004484* Int 1 (−)	Long intergenic non coding RNA
2q31.2b (+)	*MIR548* *N* Int 1 (+)^b^	Homo sapiens microRNA 548n
3q25.1b (+)	*CLRN1-AS1* Int 1 (+)	CLRN1 antisense non-coding RNA
4p13* (−)	*TCONS_00007753* Int 1 (−)	Long intergenic non coding RNA
4q23 (−)	*LOC100507053* Int 1 (+)	Uncharacterized antisense long non-coding RNA
4q28.3 (+)	*TCONS_00007833* Int 1 (−)	Long intergenic non coding RNA
4q32.3 (+)	*MIR5684* Int 2 (+)	MicroRNA involved in post-transcriptional regulation of gene expression
6q15 (−)	*TCONS_00011526* Ex 1, Int 1 (−)	Long intergenic non coding RNA
6q27a° (+)	*TCONS_l2_00024517* Int 2, Ex 3 (+) *TCONS_l2_00024518* Int 1, Ex 2 (+) *TCONS_l2_00024519* Int 1 (+)	Long intergenic non coding RNAs
7p14.2* (+)	*DQ594967* Ex 1(−)^b^	Antisense non coding RNA
8q12.1 (−)	*TCONS_00015019* Int 1 (−) *AC022555.1* (−)	Long intergenic non coding RNA Pseudogene
9p21.3 (+)	*LOC441389* Int 5 Ex 6 (+)^b^	Uncharacterized long non-coding RNA
10q11.22 (−)	*TCONS_00017977* Int 1 (−)	Long intergenic non coding RNA
11q14.2 (−)	*PRSS23* Int 2 (+)	Protease serine 23 near-coding RNA
11q23.3 (−)	*TMPRSS4-AS1* Int 2 (−)^b^	Antisense non-coding RNA
13q21.33* (+)	*LINC00383* Ex 1, Int 1 (+)	Long intergenic non coding RNA
13q31.3° (+)	*TCONS_00021873* Int 2 (+)	Long intergenic non coding RNA
21q21.1* (−)	*MIR548XHG* Ex 1, Int 1 (−)	MIRNA548X host gene long non-coding RNA
14q22.1 (+)	*AL163953.3* Int 3 (+)	Long non-coding RNA
19p12d (+)	*AK125686* Int 2 (−)^b^	Antisense non coding RNA
Xq13.3* (−)	*TCONS_00016997* Ex 1–2, Int 1 (+) *AL451105.1* (−)	Long intergenic non coding RNA Pseudogene

Proviruses and undefined sequences are labeled respectively with * and °. For HERV-W sequences and genes, the strand direction is reported into round brackets

^a^Already reported in Li et al. [70]

^b^Already reported in Schmitt et al. [20]

Finally, the HERV-W elements were evaluated for the possibility to bind cellular transcription factors (TFs) that normally interact with the host DNA. The analysis was based on the data obtained through ENCODE Transcription Factors ChIP-seq and Factorbook databases, and the TFs with higher score (ranging from 800 to 1000) were considered to be the most reliable (Table 4). Results showed that 16 HERV-W elements could tentatively bind cellular TFs, including POLR2A, MAFK, E2F1 and TCF7L2. Twelve of these elements were proviruses, and 7 of them plus 1 pseudogene were inserted into human coding genes. The higher representation of proviral sequences is probably due to the presence of complete LTR structures. Despite the fact that the detection of predicted TF binding sites is not enough to suggest a possible transcription, their presence in HERV-W elements that are co-localized with human genes could potentially have an effect on the transcription of such genes and need to be further investigated at expression level.

**Table 4:** HERV-W genomic context: transcription factor (TF) binding sites

HERV-W	TF recognized	Position	Score (0–1000)
2p12a*	POLR2A	chr2:76098843–76099352	803
2q22.2*	E2F1	chr2:143661226–143661546	958
3p22.2*	CTCF	chr3:38331061–38331485	900
4p16.1*	POLR2A	chr4:8429472–8430544	1000
	TCF7L2	chr4:8424096–8424592	922
6p12.2*	TFAP2C	chr6:52783052–52783485	1000
	FOXA2	chr6:52783244–52783462	848
	STAT3	chr6:52784270–52784579	808
6q14.3*	STAT3	chr6:85427859–85428174	1000
	CEBPB	chr6:85427862–85428118	817
7q21.1*	TCF7L2	chr7:92103429–92103733	1000
**7q31.1a**	TCF7L2	chr7:107981247–107981830	1000
	E2F1	chr7:107981308–107981897	1000
7q33*	YY1	chr7: 34270591–134271127	1000
7q36.1*	FOXA1	chr7:149370177–149370408	1000
9q22.1	TCF7L2	chr9:91556701–91556965	1000
10q21.2*	GATA3	chr10:62797340–62797529	1000
	E2F1	chr10:62796837–62797697	806
10q23.1*	GATA1	chr10:86284672–86285183	1000
	MAFK	chr10:86285572–86285911	1000
	MAFF	chr10:86285647–86285793	1000
	TBL1XR1	chr10:86284785–86285185	824
**10q24.1**	TAL1	chr2:97480654–97480806	928
	TEAD4	chr10:97480630–97480810	859
10q21.3	MAFK	chr10:65805045–65805364	1000
21q21.1*	TFAP2C	chr21:20128637–20128925	1000
	YY1	chr21:20131977–20132464	859

Data obtained from Genome Browser Encode Transcription Factor ChIP-seq database

Proviruses and undefined sequences are labeled respectively with * and °. Bold loci are the one inserted into human genes

### Env putative proteins analysis

Due to its physiological role, the ERVWE1/Syncytin-1 ORF has been characterized in detail in terms of structure and functional domains, as recently reviewed [73]. Hence we wanted to compare the Syncytin-1 precursor ORF and its features with respect to the most complete *env* genes found in our dataset, in order to predict the conservation of those sites reported to be involved in Syncytin-1 protein in vivo functions. Within our HERV-W elements, in addition to the Syncytin-1 locus ORF (7q21.2, 538 aa), we found 16 full-length or near full-length (longer than 1.4 Kb) *env* genes, 3 more than previously reported with similar parameters [74], and 10 conserved but shorter *env* genes (from 1398 to 801 nucleotides). These *env* genes manual translation led to the correspondent putative proteins (puteins), with a length range of 483–559 aa and 267–466 aa, respectively (Table 5). These *Env* puteins were obtained from different reading frames in the often-damaged *env* gene candidates, and are thus just a bioinformatics model useful to evaluate the predicted domains structure. *Env* puteins were aligned and analyzed with respect to the Syncytin-1 amino acid sequence (NCBI reference NP_055405.3), showing a general accumulation of nucleotide substitutions, insertion and deletion. This led to the frequent occurrence of multiple premature internal stop codons as well as many frameshifts, which prevents the effective production of a complete protein (Additional file 4: Fig. S3, Additional file 5: Fig. S4).

**Table 5:** Env puteins analysis

Sequence	ORF length (amino acids)	Stop	Shift
7q21.2*	**538**	**0**	**0**
1p32.3b*	559	3	2
6q21a	552	4	2
15q21.3	543	2	2
4q13.3*	542	0	2
5q11.2	542	0	4
5q21.3*	542	6	2
12q13.12*	542	2	1
14q21.2*	542	3	1
*Xq22.3b*	542	1	0
3q11.2*	541	1	3
4q31.1*	541	2	2
17q12b	540	2	2
Xp22.31	529	0	6
20q13.2	483	0	1
11p15.4	475	2	3
3p24.1*	466	2	0
9q31.3	462	3	1
3q23a	453	2	1
*4q32.3*	443	1	2
1q32.3a	361	0	3
5p12*	355	6	0
1p34.2	352	2	1
Xq27.1	352	4	1
4q21.22*	320	0	1
17q12a	296	0	2
*9q22.31*	267	1	0

Proviruses are labeled with *, Syncytin-1 ORF is highlighted in bold. Underlined sequences retain an ORF without internal stop codons; italic sequences did not present frameshifts

Noteworthy, seven *Env* puteins conserved a coding sequence without internal stop codons. Among them, three *env* genes (4q13.3, 5q11.2 and Xp22.31) are theoretically long enough to encode a complete protein (Additional file 4: Fig. S3). However, even if uninterrupted, those ORFs showed changes of reading frame with respect to the Syncytin-1 translation mode. 20q13.2 (483 aa) and 4q21.22 (320 aa) sequences are the most conserved with respect to Syncytin-1, presenting no stop codons and only one frameshift between positions 441–442 and 75–76, respectively. Xq22.3b (542 aa) and 9q22.31 (267 aa) present indeed no frameshifts but showed a single internal stop codon (position 39 and 149, respectively) that could potentially be reverted with a single point mutations, as already demonstrated ex vivo for Xq22.3b N-trenv [75].

Regarding the amino acid composition, all investigated Env puteins accumulated several substitutions, leading to a general average identity of about 85 % with respect to Syncytin-1 sequence. To evaluate the puteins possible biological activity, we have characterized in detail the motifs known to be mostly involved in the Syncytin-1 physiological function. Primarily, the envelope precursor must be processed into the mature SU and TM units, with a proteolytic cleavage that occurs at the Furin Cleavage Site conserved RKNR motif. The mutation of this conserved domain has been reported to abrogate the proteolytic cleavage and the fusogenic activity of Env proteins, that exhibited also delayed kinetics of appearance on the membrane compared to the wild-type envelope [76]. The RKNR motif of the HERV-W puteins was frequently mutated at the first position, mostly with the conversion of R residue to C or H (73 % of analyzed ORFs), but was maintained in 7 sequences. After cleavage, SU and TM mature proteins are then linked through a covalent disulphide bond between the SU CWIC and the TM CX6CC motifs. While the TM domain showed a high degree of amino acid homology with respect to Syncytin-1, in the SU motif we found an I > M substitution in 100 % of sequences. Another fundamental step that drives the fusion activity is the interaction between the SU N-terminal 124 aa receptor binding domain and a human sodium-dependent neutral amino acid transporter (hASTC1 or hASTC2), which acts as type D mammalian retrovirus receptor. In the binding domain, the SDGGGX2DX2R motif was recognized to be essential for the receptor contact, and was found in the 58 % of the sequences. The Syncytin-1 fusogenic activity is held by the TM portion, that includes a fusion peptide and a fusion core formed by the amino- and carboxy-terminal heptad repeats. In *Env* puteins the fusion peptide sequence was characterized by at least one substitution, with residue 332 (A) that was mutated in all sequences into an R or a G (and in one case into an E). Also the fusion core was affected by several mutations localized in both heptad regions, like the residue 433R > Q substitution that is present in 25 out of 26 carboxy-terminal repeats. Interestingly, the 75 amino acids long heptad repeat region showed also a higher concentration of internal stop codons, harboring 50 % of the total stop codons found in the analyzed puteins. Moreover, in traditional Env proteins the fusogenic activity is prevented by an inhibitory R peptide that is located in the TM intracytoplasmic tail and is normally removed by viral proteases. In Syncytin-1 a four amino acid deletion at the LQMV cleavage site made the protein constitutively competent for fusion [77]. This mutation was not present in any other analyzed HERV-W Env putein. Finally, the Syncytin-1 TM subunit also contains a conserved immunosuppressive domain that was thought to possibly contribute towards maternal immunotolerance [12] even though following findings suggested the absence of this activity [14]. In any case, in the selected *Env* puteins this domain presents several amino acid substitutions and in 5 sequences a premature termination at position 383. Hence, with respect to locus 7q21.2 Syncytin-1 protein, the other HERV-W loci *Env* puteins resulted highly defective, especially in sites involved in known physiological functions. However, despite these mutations, they may still be able to produce shorter proteins with a biological significance and/or a role in disease development, as observed for other HERV sequences [78].

Due to its maintenance despite the presence of huge recurrent flanking deletions affecting the 85 % of HERV-W *env* genes, also the small *env* portion of about 30 nucleotides at position 8289–8318 was translated and compared with respect to Syncytin-1. As shown in Additional file 6: Fig. S5, all the 138 HERV-W elements that maintained this portion showed recurrent amino acid substitutions. In particular, the N in position 3 was changed in 136/138 sequences, substituted by H in 93 % of the elements; while the V in position 8 was substituted in 135 sequences, showing a I in 90 % of cases. This prevalence indicates that Syncytin-1 protein probably represents the exception, suggesting an unreported functional relevance of this short domain.

### MSRV sequences homology with HERV-W elements

To complete the overview on the HERV-W presence and impact on human genome it was useful to consider also the proposed association with MS disease. In fact, the first HERV-W member was originally identified as cDNA sequences derived from particle-associated RNA in MS patients cultured cells [79, 80]. Those sequences were subsequently indicated as MS associated Retrovirus (MSRV) [11, 81, 82], proposed to be an exogenous competent member of the HERV-W group, related to the MS development [83–86]. Other reports, however, remarked the uncertain nature of MSRV [28, 87, 88] and proposed that some of these cDNA sequences could arise from the recombination of different HERV-W loci transcripts [89]. According to this hypothesis, such recombination could happen through RT switching templates, likely during in vitro PCR amplification, that is a common complication during the analyses of transcribed elements [90]. In particular, Laufer et al. proposed that six sequences, previously published as MSRV elements, could be traceable to a single HERV-W locus or a recombination between two HERV-W loci transcripts [89]. Since other four sequences published as MSRV (accession numbers: AF009668, AF009666, AF009667 and AF123880) [81, 82] were not analyzed for possible HERV-W origin, having a more complete HERV-W database we analyzed them as described [89], including the six previously investigated MRSV sequences as internal control (Table 6). The analysis confirmed that AF127227, AF127228, AF123882, and AF123881 have high identity with a single HERV-W locus, while AF127229 and AF331500 could origin from recombination of two HERV-W loci transcripts. Similarly, AF009666 pro-pol and AF009667 pol mRNAs showed high identity with the HERV-W locus 1p34.2 (99.5 % similarity) and 17q22 (98.2 % similarity), respectively, while AF123880 5′ LTR-pre gag mRNA showed sequence identity with three HERV-W loci (5p12, 3p24.1 and 3q26.32) with a similarity for each component ranging from 98 to 100 %. Finally, AF009668 pro-pol mRNA showed a more complex identity pattern with a high degree of mosaicism that seems to involve several HERV-W loci: 1p34.2, 2p12a, 2p24.2, 6q27b, 6q15 and 3p12.3. Interestingly, AF009668 shares a 95 % similarity with AF135487, a retroviral-related sequence identified to be schizophrenia associated and also mapped to multiple sites [91]. Moreover, we performed the same analysis with the four MSRV DNA probes used to characterize the HERV-W placental expression. These probes were obtained through RT-PCR from RNA particles found in synoviocyte culture supernatants and pellets of a Rheumatoid arthritis patient (AF072494 pol- and AF072498 env-probes respectively) and in B lymphocyte culture and plasma of a MS patient (AF072496 gag- and AF072497 pro-probes respectively) [11]. Both AF072494 pol- and AF072496 gag-probes showed high identity with HERV-W 6q21b locus (99.6 % similarity), while AF072497 pro- and AF072498 env-probes were highly identical to locus 1p34.2 (99.2 %) and Xq22.3b (99.5 %), respectively.

**Table 6:** HERV-W loci homology of previously described MSRV sequences and probes

MSRV GenBank entry	HERV-W locus/loci	Query cover	n° of discordant bases	Mapped portion in LTR17-HERV17-LTR17
AF127227 (544 bp)	3q23a* (99.5 %)	1–544	3	env (8208–8752)
AF127228 (1932 bp)	Xq22.3b* (99.6 %)	1–1932	9	pol-env (5444–5838 and 7682–9200)
AF127229 (2004 bp)	3p12.3* (99.9 %)	1–1084	2	pol-env-3′ LTR (5452–6792 and 8290–8318 and 9115–9732)
	18q21.32* (99.9 %)	1055–2004	2	
AF123882 (2477 bp)	12q21.3* (99.8 %)	1–2477	7	pol-env (5720–8199)
AF331500 (1629 bp)	Xq22.3b* (99.7 %)	1–1332	4	env (7720–9348)
	5p12* (99.4 %)	1308–1629	2	
AF123881 (1511 bp)	3q26.32* (99.9 %)	1–1511	2	gag-pro (2765–4269)
AF009668 (2304 bp)	1p34.2 (99.1 %)	1–633	6	pro-pol (4178–6480)
	2p12a (100 %)	623–736	0	
	2p24.2 (100 %)	717–871	0	
	6q27b (98.5 %)	837–1424	11	
	6q15 (97.2 %)	1415–1763	10	
	3p12.3 (99.4 %)	1719–2304	4	
AF009666 (324 bp)	1p34.2 (99.5 %)	1–324	3	pro-pol (4178–4521)
AF009667 (118 bp)	17q22 (98.2 %)	1–118	2	pol (5031–5148)
AF123880 (1003 bp)	5p12 (99.6 %)	1–203	1	5′ LTR (255–803)
	3p24.1 (100 %)	198–593	1	
	3q26.32 (98 %)	592–1003	11	
AF072494 pol probe (678 bp)	6q21b (99.6 %)	1–678	5	pol (4660–5338)
AF072496 gag probe (536 bp)	6q21b (99.6 %)	1–536	2	pre gag-gag(2706–3199)
AF072497 pro probe (364 bp)	1p34.2 (99.2 %)	1–364	4	pro-pol (4166–4522 and 5641–5549)
AF072498 env probe (591 bp)	Xq22.3b (99.5 %)	1–591	3	env (8606–9196)

Previously published MSRV sequences and probes (column 1) were analyzed for their homology to one/more HERV-W locus/loci by BLAT search, considering the best match in human genome (reported in column 2 near to each HERV-W element). The MSRV elements portion similar to HERV-W locus/loci (column 3) as the number of discordant nucleotides with respect to the identified HERV-W locus/loci (column 4) and the correspondent positions in the LTR17-HERV17-LTR17 reference (column 5) were obtained through Mafft alignment and Geneious platform analysis. MSRV sequences were characterized through the analysis of each element with respect to the whole HERV-W dataset with Recco software

* Already investigated by Laufer et al. [89]

° 95 % similarity with AF135487, a retroviral-related sequence reported to be schizophrenia associated and mapped to multiple sites

The MSRV sequences containing an *env* gene (or a portion of it) and showing highest identity with one of the HERV-W loci analyzed for *Env* puteins were manually translated and aligned with the correspondent HERV-W *Env* putein and the Syncytin-1 protein for further comparison (Additional file 7: Fig. S6). Interestingly, with respect to the Syncytin-1 sequence the HERV-W puteins and the correspondent MSRV putein shared the great majority of amino acid substitutions, and often the same amino acid change was common to all sequences analyzed. AF127227 and 3q23a share the same frameshift at position 270 of Syncytin-1 sequence. Moreover, AF127227 and AF127228 showed an internal stop codon at the same position observed in 3q23a and Xq22.3b, respectively (position 39, W in Syncytin-1). Differently, AF331500 lacks this internal stop codon presenting, like Syncytin-1, a W in this position. As already observed for HERV-W, also MSRV *Env* puteins showed at least one amino acid change in all domains relevant to Syncytin-1 biological activity. Given the proposed MSRV Env proteins role in pathogenesis, the presence of shared recurrent substitutions, possibly preventing the MSRV *Env* puteins functionality as compared to Syncytin-1, opens further questions that will have to be addressed. Overall, while more MSRV RNA expression studies are needed, the here reported HERV-W genomic map and characterization is a further step to properly assess the MSRV/HERV-W role in the context of MS.

## Conclusions

Since the discovery of Syncytin-1 role in placentation [11–13, 92], a great attention has been dedicated to the expression potential of the HERV-W group, trying to further understand their impact on the host. Many studies were focused on HERV-W correlations with several human diseases, primarily represented by MS [15–21, 28, 75, and reviewed in 76] and other major neurological pathologies such as schizophrenia and bipolar disorder [23, 25, 93]. Despite this broad investigation, no certain correlations between HERV-W group expression and any human disease has been confirmed. Also in the major field of MS the findings are still highly discordant [28]. One of the problems faced in this scenario is still the unfortunate lack of a complete and updated description of the HERV-W sequences in the human genome, their genomic background and a detailed knowledge of HERV-W single members. Such information could help in better interpreting the wide range of collected HERV-W expression data.

Therefore, using more updated genome data and a double bioinformatics identification approach, we performed an analysis on the GRCh37/hg19 assembly identifying a total of 213 HERV-W unambiguously classified members. Each HERV-W sequence has been precisely localized and characterized in term of structure, phylogeny and evolution, allowing to specifically identify the uniqueness of each HERW-W single member, and highlighting various non-previously reported characteristics of the group.

Firstly, we observed several nucleotide differences of HERV-W members with respect to the assembled LTR17-HERV17-LTR17 reference that was built on a small number of sequences and therefore does not properly represent the entire group. Secondly, we classified the HERV-W members into two subgroups through a LTRs phylogenetic analysis strongly supported by the identification of key mutated positions in both LTRs, shared by the majority (from 95 % up to 100 %) of sequences within the same subgroup. Beside LTRs mutations relevant for classification purposes, the subgroups comparisons showed single nucleotides differences along the whole retroviral sequence. For this reason we propose here two new consensuses, one for each subgroup (Additional file 8: File S1), that in our opinion better represent the overall HERV-W group composition.

In the present study, for the first time, the period of insertion has been estimated for each HERV-W locus through at least two different methods of age calculation. This provided a precise and exhaustive picture of the group diffusion among primates, and brought important improvements in the method reliability and applicability. Moreover, the analysis showed significantly different dynamics in the two subgroups diffusion, pointed out also by the analysis of the PBS type variability.

The analysis of structural features described for Gammaretroviruses [7] in HERV-W single members allows to characterize them for the first time in term of prevalence and sequence conservation among the group. Noteworthy, in addition to the traditional Zinc finger motif [94], we found a previously unreported second putative Zinc finger with an unusual structure, lacking one variable residue. Another interesting feature reported here for the first time is the presence of a weak bias in the HERV-W elements purine amount, with enrichment in A and a consequent underrepresentation of G.

With regards to the group genomic context, we provide an updated overview of 80 HERV-W elements inserted into human genes and the predicted capacity to bind cellular TFs. In particular, 55 HERV-Ws were found into coding genes, 8 more than what previously observed [20, 70], while 25 elements were inserted in human non-coding genes, of which the great majority (22) are reported here for the first time.

*Env* putein analysis led us to identify and functionally characterize 16 full-length or near full-length *env* genes, 3 more than previously reported [74], and 10 conserved but shorter *env* genes. Although the relative puteins resulted highly defective and mutated in comparison to Syncytin-1, these genes may still be able to produce shorter proteins with a biological significance, as observed for other HERV sequences [78].

In the light of the debated connection between HERV-W loci expression and MS disease, we investigated the elements known as MSRV in order to evaluate their identity with respect to one or more HERV-W loci in agreement to what has been previously reported [89]. Our results confirmed that the majority of MSRV related sequences have from 97 to 100 % identity with one single HERV-W locus, but more complex pattern of identity, apparently involving 3 or even 6 loci, were also observed. Furthermore, the comparison between MSRV *Env* puteins and the highest identical HERV-W loci puteins showed common amino acid substitutions with respect to Syncytin-1, that affect all domains reported as relevant for its biological activity.

In conclusion, this report provides, to our knowledge, the most exhaustive and updated overview to date on HERV-W group in terms of structure, evolution and context of integration into the human genome, revealing that this polymorphic multicopy family is not only represented by the single HERV-W member Syncytin-1. We showed that HERV-W elements were acquired by primates during a rather long period, and evolved within and with their genome that exerted a selective pressure leading to the modification of HERV-W structures, including the previously shown co-option of one member for an important physiological function [12, 13]. Overall, the here presented characterization of the HERV-W composition and their genomic context of insertion, will be essential to investigate the effects that, beside protein expression, HERV-W can exert in different tissues both in physiological conditions as well as putative involvement in human disease development and clinical manifestations and to better define their real impact and contribution to our genome.

## Methods

### HERV-W identification and localization

The 213 HERV-W sequences were collected from GRCh37/hg19 assembly using a double approach that binds (1) the hg19 assembly analysis by the ReTe program package [50] and (2) a traditional BLAT search [51] in the UCSC Genome Browser database [95] using the RepBase Update [52] assembled LTR17-HERV17-LTR17 consensus as a query. The elements found by both approaches have then been confirmed as HERV-W based on (1) Repeat Masker analysis of the HERV-W sequence and its genomic flanking portions, (2) structural alignment and comparison with respect to the HERV-W group RepBase reference LTR17-HERV17-LTR17 and (3) phylogenetic trees; in order to avoid misclassifications or incomplete sequences inclusion.

HERV-W solitary LTRs were retrieved by UCSC Genome Browser BLAT search using LTR17 as a query, and kindly provided by Professor Jens Mayer (Saarland University).

### Sequences alignment and structural characterization

The HERV-W nucleotide composition was characterized in detail with respect to the RepBase Update assembled LTR17-HERV17-LTR17 reference by multiple alignments performed with Mafft on line program, version 7 [96] and the subsequent analysis on Geneious bioinformatics software platform, version 8.1.4 [97]. All insertion and deletions were annotated, and the presence of other repetitive elements was reported.

### Phylogenetic trees

Phylogenetic trees were built with Mega Software, version 6 [98] using pairwise deletion and p-distance method with 500 bootstrap replications. In addition to HERV-W nucleotidic sequences and RepBase Update LTR17 and HERV17 consensus, each tree initially included a HERV9 generated consensus [5]. This was initially made in order to identify and eliminate eventual members of this HERV-W related family.

### Time of integration estimation

The age of the single HERV-W members was estimated based on the percentage of divergent nucleotides (D %) between (1) 5′- and 3′ LTRs of each provirus, (2) proviral and pseudogenic single LTRs and a generated consensus for each subgroup, and (3) proviral and pseudogenic 150–300 nucleotides *gag*, *pro*, *pol* RT, *pol* IN and *env* portions and a generated consensus for each subgroup. The divergence values were estimated on Mega 6 through Kimura 2-parameter corrected pairwise distances excluding gaps and CpG dinucleotides. The D % have then been used according to previous methodologies [56] to estimate the time of integration (T) assuming an human genome substitution rate of 0.13 %/nucleotides/million years, with the formula T = D/0.13. For the proviral 5′- versus 3′ LTR divergence a factor of 2 was applied assuming that each LTR evolved independently into the genome (T = D/0.13/2). The final age of each sequence was expressed as average of the estimated time of integration obtained, excluding those value with a standard deviation >20 %.

### PBS and gammaretroviral features representation

The presence and composition of the PBS nucleotide sequence and of the nucleocapsidic Zinc finger and C-terminal polymerase IN GPY/F amino acid motifs were analyzed using Mafft alignment and Geneious platform. The grade of conservation at each position was represented with a logo built from WebLogo at http://weblogo.berkeley.edu [99]. The PBS assignation to the correspondent human tRNA type was made by similarity analysis with respect to a tRNA library built from the Transfer RNA database (tRNAdb) of Leipzig University [100] and from the PBS library provided by Professor Jonas Blomberg [5].

### Genomic context

The genomic context of each HERV-W sequence was characterized by the integration of their genomic coordinates with the UCSC Genome Browser Genes and Genes prediction tracks [101–103]. The elements co-localized with human genes were further analyzed by BLAST search after the activation of OMIM, UCSC, RefSeq and Gencode genes annotations [104]. The presence of TFs binding sites were characterized by the integration of HERV-W members genomic coordinates with the UCSC Genome Browser Regulation Encode Txn Factor ChIp tracks [105, 106]. TFs binding sites were considered reliable in the presence of a score ranging from 800 to 1000.

### Env puteins analysis

The *env* selected genes were translated in all possible frames using Geneious platform. The alignment with respect to ERVWE1/Syncytin-1 precursor (NCBI reference sequence NP_055405.3) was performed on Mafft and allowed to reconstruct the complete protein and to annotate all frameshifts and stop codons. The structural and functional relevant domains were analyzed on Geneious platform.

### Analysis of MSRV sequences

Previously published MSRV sequences and probes were retrieved from GeneBank and analyzed by BLAT search for the best matching HERV-W locus/loci based on nucleotide sequence similarity in GRCh37/hg19 assembly. Alignments of MSRV sequences and the relative best matching HERV-W elements were manually inspected on Geneious platform, and discordant positions were annotated. The HERV-W locus/loci homology was then confirmed through the software Recco [107] with respect to our whole HERV-W dataset as described [89].

### HERV-W consensus sequences generation

The HERV-W group and subgroups consensus sequences were generated from our HERV-W dataset using Geneious bioinformatics software platform, version 8.1.4 [97].

## Additional files

10.1186/s12977-016-0301-x HERV-W elements identification in Human Genome assembly GRch37/hg19.
10.1186/s12977-016-0301-x Phylogenetic neighbor joining trees of proviral and pseudogenic (A) *gag*, (B) *pol* and (C) *env* genes. RepBase HERV17 consensus is labeled with a black square.
10.1186/s12977-016-0301-x Phylogenetic neighbor joining trees of about 800 HERV-W LTRs retrieved from GRCh37/hg19 assembly. LTR17 consensus is labeled with a black square.
10.1186/s12977-016-0301-x Env puteins analysis with respect to Syncytin-1 (locus 7q21.2). In Syncytin-1 ORF the domains mostly involved in the protein structure and function are annotated: Leader Peptide (LP), Binding Domain motif (BD), SU and TM disulphide bounds motifs (dS), Furin cleavage site (FCS), fusion core N- and C- terminal Heptad Repeats (NHR and CHR), Immunosuppressive domain (IM), Transmembrane unit (TM), Intracytoplasmic Tail (CYT) and the relative Syncytin-1 specific deletion (LQMV del). In Env puteins amino acid substitutions and internal stop codons with respect to Syncytin-1 are labeled with colored and black lines, respectively. The reading frames are reported below each sequence with a number and an arrow.
10.1186/s12977-016-0301-x Residues-visible version of Env puteins analysis with respect to Syncytin-1 (locus 7q21.2). In Syncytin-1 ORF the domains mostly involved in the protein structure and function are annotated: Leader Peptide (LP), Binding Domain motif (BD), SU and TM disulphide bounds motifs (dS), Furin cleavage site (FCS), fusion core N- and C- terminal Heptad Repeats (NHR and CHR), Immunosuppressive domain (IM), Transmembrane unit (TM), Intracytoplasmic Tail (CYT) and the relative Syncytin-1 specific deletion (LQMV del). In Env puteins amino acid substitutions and internal stop codons with respect to Syncytin-1 are labeled with colored and black squares, respectively. The reading frames are reported below each sequence with a number and an arrow. The orange arrow indicates the Env portion frequently maintained in presence of flanking huge recurrent deletions.
10.1186/s12977-016-0301-x HERV-W Env puteins maintained portion comparison with respect to Syncytin-1 ammino acid sequence. Amino acid substitutions are labeled with colored and squares. A consensus sequence is shown above Syncytin-1.
10.1186/s12977-016-0301-x MSRV Env puteins analysis with respect to the most similar HERV-W loci Env puteins and Syncytin-1 (locus 7q21.2). In Syncytin-1 ORF the domains mostly involved in the protein structure and function are annotated: Leader Peptide (LP), Binding Domain motif (BD), SU and TM disulphide bounds motifs (dS), Furin cleavage site (FCS), fusion core N- and C- terminal Heptad Repeats (NHR and CHR), Immunosuppressive domain (IM), Transmembrane unit (TM), Intracytoplasmic Tail (CYT) and the relative Syncytin-1 specific deletion (LQMV del). In Env puteins amino acid substitutions and internal stop codons with respect to Syncytin-1 are labeled with colored and black squares, respectively. The reading frames are reported below each sequence with a number and an arrow. The orange arrow indicates the Env portion frequently maintained in presence of flanking huge recurrent deletions.
10.1186/s12977-016-0301-x HERV-W proposed consensus in Fasta format. HERV-W group general proviral consensus sequence (HERVW_PVcons) and HERV-W subgroups 1 and 2 consensus sequences (HERVW_SG1cons and HERVW_SG2cons, respectively) generated starting from our HERV-W sequences dataset.
