# Supplementary material for: Contribution of type W human endogenous retroviruses to the human genome: characterization of HERV-W proviral insertions and processed pseudogenes
Source: Retrovirology. 2016 Sep 9;13(1):67. doi: 10.1186/s12977-016-0301-x (PMC5016936; doi:10.1186/s12977-016-0301-x)
Supplement: Supplementary file 1 — 10.1186/s12977-016-0301-x HERV-W elements identification in Human Genome assembly GRch37/hg19. [file 12977_2016_301_MOESM1_ESM.docx]

| **Table S1. HERV-W elements identification in Human Genome assembly GRch37/hg19**   \| **Locus** \| **Strand** \| **Start** \| **End** \| **Type** \| **Subgroup^a^** \| **PBS type** \| **O.C.A^b^** \| **Age^c^** \| \| --- \| --- \| --- \| --- \| --- \| --- \| --- \| --- \| --- \| \| 1p34.2 \| + \| 42410127 \| 42415982 \| pseudogene \| 1 \| -^d^ \| Rhesus \| 32,52 \| \| 1p33a \| - \| 46851453 \| 46856995 \| pseudogene \| 1 \| W \| Gibbon \| 26,99 \| \| 1p33b \| + \| 47417563 \| 47424579 \| pseudogene \| 1 \| W \| Rhesus \| 33,82 \| \| 1p32.3a \| + \| 51692797 \| 51696169 \| pseudogene \| 2 \| - \| Rhesus \| 30,11 \| \| 1p32.3b \| - \| 55376682 \| 55385198 \| provirus \| 1 \| W \| Rhesus* \| 26,82 \| \| 1p32.2 \| - \| 56248856 \| 56254641 \| provirus \| 2A \| F \| Rhesus \| 32,99 \| \| 1p22.2a \| + \| 89390212 \| 89397564 \| pseudogene \| 1 \| W \| Rhesus \| 32,09 \| \| 1p22.2b \| + \| 91644689 \| 91646698 \| pseudogene \| 1 \| - \| Rhesus \| 24,73 \| \| 1p13.3 \| - \| 110394855 \| 110400764 \| provirus \| 1 \| - \| Orangutan \| 29,30 \| \| 1p12 \| - \| 119710922 \| 119713987 \| pseudogene \| 1 \| - \| Rhesus \| 26,34 \| \| 1q22 \| - \| 155592180 \| 155596455 \| undefined \| - \| - \| Orangutan \| - \| \| 1q25.2 \| - \| 178110353 \| 178112194 \| pseudogene \| 2 \| - \| Rhesus \| 39,24 \| \| 1q32.1 \| - \| 205835460 \| 205840608 \| pseudogene \| 2 \| - \| Rhesus \| 36,76 \| \| 1q32.3a \| - \| 212029138 \| 212031215 \| pseudogene \| 1 \| - \| Rhesus \| 31,32 \| \| 1q32.3b \| + \| 212031216 \| 212032263 \| undefined \| - \| - \| Rhesus \| - \| \| 1q42.13 \| - \| 227812215 \| 227821260 \| provirus \| 2A \| F \| Rhesus \| 39,88 \| \| 2p25.3 \| - \| 153394 \| 160782 \| provirus \| 1 \| W \| Rhesus* \| 24,45 \| \| 2p24.2 \| + \| 17520208 \| 17527981 \| provirus \| 1 \| R \| Gibbon \| 23,94 \| \| 2p23.1a \| + \| 30738819 \| 30743130 \| provirus \| 2A \| F \| Rhesus \| 39,00 \| \| 2p23.1b \| + \| 31854223 \| 31859227 \| pseudogene \| 1 \| R \| Orangutan \| 35,12 \| \| 2p22.3 \| + \| 33882489 \| 33889522 \| pseudogene \| 1 \| R \| Rhesus \| 35,90 \| \| 2p16.2 \| + \| 53983776 \| 53988563 \| pseudogene \| 2 \| W \| Rhesus \| 39,45 \| \| 2p12a \| + \| 76098816 \| 76106624 \| provirus \| 1 \| W \| Gibbon \| 30,23 \| \| 2p12b \| - \| 79390297 \| 79397477 \| pseudogene \| 1 \| R \| Rhesus \| 25,22 \| \| 2q11.2 \| - \| 96882882 \| 96889063 \| pseudogene \| 2 \| W \| Gibbon \| 31,15 \| \| 2q12.2 \| + \| 106825670 \| 106832132 \| pseudogene \| 1 \| W \| Rhesus \| 19,27 \| \| 2q13 \| - \| 112796923 \| 112802566 \| pseudogene \| 1 \| W \| Rhesus \| 35,18 \| \| 2q22.1 \| - \| 136959456 \| 136960929 \| pseudogene \| 1 \| - \| Gibbon \| 40,75 \| \| 2q22.2 \| - \| 143656248 \| 143665468 \| provirus \| 2B \| N \| Rhesus \| 45,10 \| \| 2q22.3 \| + \| 147756109 \| 147758691 \| undefined \| - \| R \| Rhesus \| - \| \| 2q24.3 \| - \| 165514421 \| 165516121 \| pseudogene \| 1 \| - \| Gibbon \| 23,59 \| \| 2q31.1a \| - \| 172416543 \| 172418614 \| pseudogene \| 1 \| - \| Gibbon \| 49,74 \| \| 2q31.1b \| + \| 176189870 \| 176191257 \| pseudogene \| 1 \| - \| Gibbon \| 39,76 \| \| 2q31.2a \| - \| 178273969 \| 178277886 \| pseudogene \| 2 \| W \| Rhesus \| 39,66 \| \| 2q31.2b \| + \| 179327137 \| 179328271 \| pseudogene \| 1 \| - \| Gibbon \| 36,14 \| \| 2q31.3 \| + \| 181096404 \| 181104046 \| provirus \| 2A \| I \| Rhesus \| 42,88 \| \| 2q32.3 \| - \| 196199400 \| 196204796 \| pseudogene \| 2 \| P \| Rhesus \| 38,56 \| \| 2q35 \| - \| 218455569 \| 218457491 \| pseudogene \| 1 \| - \| Rhesus \| 31,16 \| \| 2q37.3 \| - \| 239902211 \| 239904111 \| pseudogene \| 1 \| - \| Rhesus \| 24,56 \| \| 3p24.3 \| - \| 19913287 \| 19918128 \| pseudogene \| 1 \| W \| Gibbon \| 24,16 \| \| 3p24.1 \| + \| 27042514 \| 27047921 \| provirus \| 1 \| W \| Gibbon \| 28,64 \| \| 3p22.2 \| - \| 38330909 \| 38338816 \| provirus \| 1 \| R \| Rhesus \| 30,01 \| \| 3p22.1 \| + \| 39699867 \| 39701564 \| pseudogene \| 1 \| - \| Rhesus \| 22,10 \| \| 3p21.31 \| - \| 48372016 \| 48377731 \| pseudogene \| 2 \| ?^e^ \| Rhesus \| 42,89 \| \| 3p12.3 \| - \| 74921984 \| 74927237 \| pseudogene \| 1 \| R \| Rhesus \| 35,77 \| \| 3p11.1 \| - \| 87989983 \| 87995529 \| pseudogene \| 1 \| W \| Gibbon \| 25,93 \| \| 3q11.2 \| - \| 96385297 \| 96394288 \| provirus \| 1 \| W \| Gibbon \| 28,33 \| \| 3q13.31 \| + \| 115463422 \| 115469539 \| pseudogene \| 2 \| - \| Rhesus \| 35,56 \| \| 3q13.32 \| + \| 118561539 \| 118570538 \| undefined \| - \| - \| Rhesus \| - \| \| 3q22.1 \| - \| 130915705 \| 130918941 \| pseudogene \| 1 \| - \| Orangutan \| 22,17 \| \| 3q22.2 \| + \| 135654191 \| 135657750 \| pseudogene \| 1 \| - \| Gibbon \| 21,10 \| \| 3q23a \| + \| 141538627 \| 141540355 \| pseudogene \| 1 \| - \| Orangutan \| 20,93 \| \| 3q23b \| + \| 142157021 \| 142162495 \| pseudogene \| 1 \| - \| Gibbon \| 25,05 \| \| 3q25.1a \| - \| 149474249 \| 149476612 \| pseudogene \| 1 \| - \| Rhesus \| 27,41 \| \| 3q25.1b \| + \| 150572043 \| 150579124 \| pseudogene \| 1 \| W \| Rhesus \| 21,75 \| \| 3q25.2 \| + \| 154695864 \| 154697090 \| pseudogene \| 1 \| - \| Gibbon \| 59,38 \| \| 3q26.1a \| + \| 162040467 \| 162046157 \| pseudogene \| 1 \| - \| Gibbon \| 27,84 \| \| 3q26.1b \| - \| 163412265 \| 163418737 \| provirus \| 2A \| - \| Rhesus \| 51,85 \| \| 3q26.31 \| + \| 172438594 \| 172441229 \| undefined \| - \| - \| Rhesus \| - \| \| 3q26.32 \| + \| 178772379 \| 178777943 \| pseudogene \| 1 \| W \| Rhesus \| 27,61 \| \| 3q28 \| + \| 191376573 \| 191383381 \| pseudogene \| 1 \| W \| Gibbon \| 25,48 \| \| 4p16.3 \| - \| 185552 \| 190863 \| provirus \| 2A \| W \| Rhesus \| 36,18 \| \| 4p16.1 \| + \| 8422092 \| 8429492 \| provirus \| 2A \| ? \| Rhesus \| 46,40 \| \| 4p15.1 \| + \| 33743127 \| 33744972 \| provirus \| 1 \| - \| Gibbon \| 52,41 \| \| 4p14 \| - \| 36442153 \| 36445845 \| pseudogene \| 2 \| - \| Rhesus \| 39,49 \| \| 4p13 \| - \| 42287455 \| 42294913 \| provirus \| 1 \| W \| Rhesus \| 29,50 \| \| 4q13.1 \| + \| 63836735 \| 63843478 \| provirus \| 2A \| E \| Rhesus \| 46,58 \| \| 4q13.3 \| - \| 73791293 \| 73798903 \| provirus \| 1 \| W \| Gibbon \| 28,59 \| \| 4q21.22 \| + \| 83394470 \| 83401206 \| provirus \| 1 \| R \| Rhesus \| 35,60 \| \| 4q21.23 \| - \| 86273279 \| 86276547 \| pseudogene \| 1 \| W \| Rhesus \| 28,85 \| \| 4q23 \| - \| 100025890 \| 100031105 \| pseudogene \| 2 \| F \| Gibbon \| 23,95 \| \| 4q24 \| + \| 106408946 \| 106410359 \| undefined \| - \| - \| Rhesus \| - \| \| 4q25 \| - \| 111200009 \| 111205600 \| pseudogene \| 1 \| W \| Rhesus \| 28,28 \| \| 4q26 \| + \| 114965536 \| 114972972 \| pseudogene \| 1 \| W \| Gibbon \| 26,84 \| \| 4q28.3 \| + \| 133803451 \| 133810295 \| pseudogene \| 2 \| W \| Rhesus \| 42,06 \| \| 4q31.1 \| - \| 139542941 \| 139548353 \| provirus \| 1 \| - \| Rhesus \| 32,78 \| \| 4q31.3 \| + \| 153762980 \| 153763482 \| pseudogene \| 1 \| - \| Rhesus \| 34,79 \| \| 4q32.3 \| + \| 165576784 \| 165579304 \| pseudogene \| 1 \| - \| Rhesus \| 46,94 \| \| 4q33 \| - \| 171111343 \| 171118255 \| pseudogene \| 2 \| W \| Rhesus \| 37,72 \| \| 4q35.1 \| + \| 183920546 \| 183925533 \| pseudogene \| 1 \| - \| Gibbon \| 18,01 \| \| 5p13.3 \| - \| 31395287 \| 31397921 \| undefined \| - \| - \| Gibbon \| - \| \| 5p13.2 \| - \| 36328801 \| 36333224 \| pseudogene \| 1 \| R \| Rhesus \| 30,76 \| \| 5p12 \| + \| 44111190 \| 44115179 \| provirus \| 1 \| W \| Gibbon \| 22,10 \| \| 5q11.2 \| - \| 56815850 \| 56818716 \| pseudogene \| 1 \| - \| Rhesus \| 27,16 \| \| 5q12.1 \| + \| 59954322 \| 59962288 \| provirus \| 2B \| I \| Rhesus* \| 28,76 \| \| 5q14.3a \| - \| 87472587 \| 87473700 \| pseudogene \| 1 \| - \| Gibbon \| 23,30 \| \| 5q14.3b \| + \| 89089703 \| 89090854 \| provirus \| 1 \| - \| Gibbon \| 62,97 \| \| 5q21.3 \| - \| 107909807 \| 107914283 \| provirus \| 1 \| - \| Gibbon* \| 25,28 \| \| 5q22.2 \| + \| 111779389 \| 111787195 \| provirus \| 1 \| R \| Orangutan* \| 20,46 \| \| 6p25.3 \| - \| 1330281 \| 1331849 \| pseudogene \| 1 \| - \| Orangutan \| 36,94 \| \| 6p23 \| + \| 13878567 \| 13883955 \| pseudogene \| 2 \| P \| Rhesus \| 32,91 \| \| 6p22.3 \| - \| 24676916 \| 24683328 \| provirus \| 1 \| W \| Gibbon \| 23,31 \| \| 6p12.2 \| - \| 52779674 \| 52786712 \| provirus \| 1 \| R \| Rhesus* \| 37,19 \| \| 6q12 \| + \| 65714885 \| 65721860 \| pseudogene \| 1 \| W \| Gibbon \| 29,41 \| \| 6q14.1 \| + \| 82046663 \| 82053574 \| provirus \| 1 \| W \| Gibbon* \| 34,15 \| \| 6q14.2 \| + \| 84153479 \| 84160109 \| pseudogene \| 1 \| W \| Rhesus \| 40,08 \| \| 6q14.3a \| - \| 85421420 \| 85430868 \| provirus \| 2A \| N \| Rhesus \| 35,48 \| \| 6q14.3b \| + \| 85653935 \| 85655028 \| pseudogene \| 1 \| - \| Orangutan \| 29,31 \| \| 6q15 \| - \| 89123912 \| 89131226 \| pseudogene \| 1 \| W \| Gibbon \| 24,15 \| \| 6q21a \| + \| 106676012 \| 106683689 \| pseudogene \| 1 \| R \| Rhesus \| 29,31 \| \| 6q21b \| + \| 107620616 \| 107621947 \| undefined \| - \| - \| Rhesus \| - \| \| 6q21c \| + \| 111452221 \| 111459275 \| pseudogene \| 2 \| S \| Rhesus \| 43,50 \| \| 6q23.3 \| - \| 138040065 \| 138043839 \| pseudogene \| 1 \| - \| Rhesus* \| 32,97 \| \| 6q24.2a \| - \| 143400938 \| 143408846 \| pseudogene \| 2 \| F \| Rhesus \| 30,65 \| \| 6q24.2b \| - \| 144437462 \| 144442702 \| pseudogene \| 1 \| W \| Rhesus \| 24,99 \| \| 6q27a \| + \| 166727385 \| 166729870 \| undefined \| - \| - \| Rhesus* \| - \| \| 6q27b \| + \| 167304571 \| 167311741 \| pseudogene \| 1 \| W \| Rhesus \| 30,55 \| \| 7p21.3 \| - \| 12892571 \| 12893302 \| pseudogene \| 2 \| - \| Rhesus \| 42,64 \| \| 7p21.1 \| - \| 16716694 \| 16717518 \| pseudogene \| 1 \| - \| Rhesus \| 39,87 \| \| 7p14.2 \| + \| 35735748 \| 35736627 \| provirus \| 1 \| - \| Rhesus \| 60,98 \| \| 7p14.1 \| - \| 40207037 \| 40213362 \| provirus \| 2B \| R \| Rhesus \| 33,16 \| \| 7q21.2 \| - \| 92097313 \| 92107506 \| provirus \| 1 \| W \| Rhesus \| 22,56 \| \| 7q31.1a \| + \| 107977725 \| 107984934 \| pseudogene \| 1 \| W \| Rhesus \| 27,33 \| \| 7q31.1b \| - \| 114019143 \| 114026368 \| pseudogene \| 1 \| W \| Gibbon \| 29,70 \| \| 7q31.31 \| + \| 119206200 \| 119210883 \| pseudogene \| 1 \| - \| Gibbon \| 29,07 \| \| 7q31.32 \| + \| 121811709 \| 121817649 \| provirus \| 2B \| - \| Rhesus \| 49,00 \| \| 7q32.3 \| - \| 131505601 \| 131509683 \| provirus \| 2A \| - \| Rhesus \| 34,32 \| \| 7q33 \| + \| 134270175 \| 134277767 \| provirus \| 2A \| F \| Rhesus \| 31,40 \| \| 7q36.1 \| + \| 149368691 \| 149374312 \| provirus \| 2B \| ? \| Rhesus \| 37,57 \| \| 8p21.3 \| + \| 20012257 \| 20017145 \| pseudogene \| 2 \| F \| Rhesus \| 45,74 \| \| 8q11.21 \| - \| 49148502 \| 49151947 \| pseudogene \| 1 \| - \| Orangutan \| 18,77 \| \| 8q12.1 \| - \| 61331973 \| 61338078 \| pseudogene \| 2 \| I \| Rhesus \| 27,03 \| \| 8q12.3a \| - \| 63507960 \| 63509929 \| pseudogene \| 1 \| - \| Gibbon* \| 17,69 \| \| 8q12.3b \| + \| 65675513 \| 65680917 \| pseudogene \| 2 \| L \| Rhesus \| 31,16 \| \| 8q13.2 \| + \| 68834197 \| 68835866 \| pseudogene \| 1 \| - \| Rhesus \| 16,94 \| \| 8q21.11 \| + \| 74734099 \| 74734838 \| pseudogene \| 1 \| - \| Gibbon \| 34,35 \| \| 8q21.13 \| + \| 81651951 \| 81655427 \| pseudogene \| 1 \| - \| Rhesus \| 28,96 \| \| 8q24.13 \| - \| 125912007 \| 125919468 \| provirus \| 2A \| ? \| Rhesus \| 32,19 \| \| 9p24.1 \| + \| 8747223 \| 8748523 \| pseudogene \| 1 \| - \| Gibbon \| 25,00 \| \| 9p21.3 \| + \| 22823028 \| 22823838 \| pseudogene \| 1 \| - \| Rhesus \| 60,62 \| \| 9p21.1 \| - \| 29656373 \| 29658925 \| undefined \| - \| - \| Rhesus \| - \| \| 9p13.3 \| - \| 35640305 \| 35642827 \| provirus \| 1 \| - \| Gibbon \| 25,06 \| \| 9q22.1 \| - \| 91554926 \| 91559339 \| pseudogene \| 1 \| R \| Rhesus \| 29,73 \| \| 9q22.31 \| + \| 94742841 \| 94744363 \| pseudogene \| 1 \| - \| Rhesus \| 40,89 \| \| 9q31.3 \| + \| 114098689 \| 114100459 \| pseudogene \| 1 \| - \| Gibbon \| 20,01 \| \| 10p12.2 \| - \| 23585121 \| 23591090 \| pseudogene \| 1 \| R \| Gibbon \| 28,02 \| \| 10q11.22 \| - \| 49873340 \| 49875049 \| pseudogene \| 1 \| - \| Gibbon \| 27,58 \| \| 10q21.2 \| - \| 62793461 \| 62799671 \| provirus \| 2A \| W \| Rhesus \| 31,53 \| \| 10q21.3 \| + \| 65804516 \| 65805376 \| pseudogene \| 1 \| - \| Gibbon \| 32,31 \| \| 10q23.1 \| - \| 86285127 \| 86290825 \| provirus \| 1 \| R \| Rhesus \| 32,39 \| \| 10q23.33 \| - \| 96594327 \| 96601288 \| pseudogene \| 1 \| R \| Gibbon \| 28,63 \| \| 10q24.1 \| - \| 97477116 \| 97484627 \| pseudogene \| 2 \| W \| Rhesus \| 29,80 \| \| 11p15.4 \| - \| 9369352 \| 9371164 \| pseudogene \| 1 \| - \| Rhesus \| 34,47 \| \| 11p14.3a \| - \| 22334363 \| 22339865 \| provirus \| 1 \| W \| Gibbon \| 31,04 \| \| 11p14.3b \| + \| 26023135 \| 26027273 \| provirus \| - \| - \| Rhesus \| 32,28 \| \| 11p14.2 \| - \| 26611978 \| 26619222 \| provirus \| 2B \| G \| Rhesus \| 29,72 \| \| 11p12 \| + \| 38623139 \| 38628790 \| provirus \| 2A \| F \| Rhesus \| 41,84 \| \| 11q14.1 \| - \| 77569373 \| 77574526 \| pseudogene \| 1 \| W \| Rhesus \| 27,24 \| \| 11q14.2 \| - \| 86544534 \| 86547860 \| pseudogene \| 1 \| - \| Rhesus \| 29,71 \| \| 11q22.3 \| - \| 107851810 \| 107853843 \| pseudogene \| 1 \| - \| Gibbon \| 39,44 \| \| 11q23.3 \| - \| 117907500 \| 117908549 \| pseudogene \| 1 \| - \| Rhesus \| 43,37 \| \| 12p13.31a \| + \| 7333743 \| 7339129 \| pseudogene \| 2 \| F \| Rhesus \| 37,45 \| \| 12p13.31b \| - \| 8914006 \| 8921050 \| pseudogene \| 2 \| W \| Rhesus \| 34,30 \| \| 12p11.1 \| + \| 34253056 \| 34255891 \| pseudogene \| 1 \| - \| Gorilla \| 25,35 \| \| 12q12a \| + \| 38865383 \| 38868227 \| pseudogene \| 1 \| - \| Gibbon \| 23,33 \| \| 12q12b \| + \| 39030303 \| 39035353 \| pseudogene \| 2 \| ? \| Gibbon \| 28,41 \| \| 12q12c \| + \| 40777438 \| 40783340 \| provirus \| 2A \| ? \| Rhesus \| 34,25 \| \| 12q13.12 \| - \| 51296259 \| 51307150 \| provirus \| 1 \| W \| Rhesus* \| 34,11 \| \| 12q13.3 \| - \| 57360856 \| 57362378 \| undefined \| - \| - \| Homo \| - \| \| 12q14.1 \| - \| 59245647 \| 59253534 \| provirus \| 1 \| W \| Gibbon \| 23,29 \| \| 12q21.31 \| + \| 85187760 \| 85192453 \| pseudogene \| 2 \| ? \| Rhesus \| 28,96 \| \| 12q23.3 \| + \| 105337032 \| 105337822 \| pseudogene \| 1 \| - \| Rhesus \| 36,98 \| \| 12q24.31 \| + \| 124032964 \| 124043310 \| provirus \| 2A \| I \| Rhesus \| 33,77 \| \| 12q24.33 \| - \| 132357772 \| 132365497 \| provirus \| 1 \| R \| Rhesus \| 35,32 \| \| 13q13.3 \| + \| 37530787 \| 37533118 \| pseudogene \| 2 \| - \| Rhesus \| 34,09 \| \| 13q21.1 \| + \| 55627766 \| 55635877 \| provirus \| 1 \| R \| Gibbon \| 19,09 \| \| 13q21.31 \| - \| 65279823 \| 65282432 \| pseudogene \| 1 \| - \| Rhesus \| 41,48 \| \| 13q21.33 \| + \| 69795752 \| 69799468 \| provirus \| 1 \| W \| Gibbon \| 39,06 \| \| 13q31.1 \| + \| 83031689 \| 83034708 \| pseudogene \| 1 \| W \| Gibbon \| 25,74 \| \| 13q31.3 \| + \| 93693066 \| 93695685 \| undefined \| - \| - \| Rhesus \| - \| \| 14q11.2 \| + \| 22704447 \| 22712069 \| provirus \| 1 \| S \| Gibbon* \| 24,00 \| \| 14q12 \| + \| 26728928 \| 26730215 \| provirus \| 1 \| - \| Gibbon \| 32,55 \| \| 14q21.2 \| - \| 45488688 \| 45492897 \| provirus \| - \| - \| Rhesus \| 36,49 \| \| 14q22.1 \| + \| 53828360 \| 53829723 \| pseudogene \| 1 \| - \| Gibbon \| 22,61 \| \| 14q23.1 \| + \| 58588999 \| 58592862 \| pseudogene \| 1 \| - \| Rhesus \| 33,96 \| \| 14q32.11 \| - \| 91692008 \| 91693212 \| pseudogene \| 1 \| - \| Gibbon \| 22,43 \| \| 15q21.3 \| - \| 55597080 \| 55604574 \| pseudogene \| 1 \| W \| Rhesus \| 26,32 \| \| 15q22.32 \| - \| 67261563 \| 67268962 \| pseudogene \| 1 \| R \| Rhesus \| 36,02 \| \| 15q26.1 \| + \| 92841377 \| 92845430 \| pseudogene \| 1 \| W \| Rhesus \| 40,53 \| \| 17q12a \| + \| 33878307 \| 33879612 \| pseudogene \| 1 \| - \| Orangutan \| 23,10 \| \| 17q12b \| + \| 35689888 \| 35693622 \| pseudogene \| 1 \| - \| Rhesus \| 35,26 \| \| 17q21.33 \| - \| 48476362 \| 48483068 \| provirus \| 1 \| W \| Rhesus \| 33,89 \| \| 17q22 \| - \| 53088886 \| 53095859 \| pseudogene \| 1 \| W \| Rhesus \| 41,51 \| \| 18p11.31 \| + \| 4681680 \| 4692409 \| provirus \| 1 \| S \| Orangutan \| 31,11 \| \| 18p11.21 \| + \| 13656678 \| 13657837 \| pseudogene \| 1 \| - \| Rhesus \| 21,27 \| \| 18q21.32 \| + \| 58377841 \| 58380952 \| pseudogene \| 1 \| - \| Gibbon \| 17,21 \| \| 18q21.33 \| + \| 60149574 \| 60150323 \| pseudogene \| 1 \| - \| Gibbon \| 25,04 \| \| 19p12a \| + \| 20210345 \| 20212271 \| provirus \| 2 \| - \| Gorilla \| 58,42 \| \| 19p12b \| + \| 21812187 \| 21817584 \| pseudogene \| 1 \| W \| Gorilla \| 22,90 \| \| 19p12c \| + \| 22928203 \| 22934876 \| pseudogene \| 1 \| W \| Gibbon \| 25,59 \| \| 19p12d \| + \| 24129884 \| 24130952 \| pseudogene \| 1 \| - \| Rhesus \| 29,50 \| \| 19q13.2a \| + \| 40577847 \| 40578561 \| pseudogene \| 1 \| - \| Rhesus \| 36,86 \| \| 19q13.2b \| - \| 41492147 \| 41494054 \| provirus \| 2 \| - \| Rhesus \| 62,29 \| \| 20p13 \| - \| 171333 \| 174808 \| pseudogene \| 1 \| - \| Gibbon \| 26,76 \| \| 20q13.2 \| + \| 53965519 \| 53969590 \| pseudogene \| 1 \| - \| Gibbon \| 30,15 \| \| 21q21.1 \| - \| 20125060 \| 20132866 \| provirus \| 1 \| W \| Gibbon \| 24,54 \| \| 21q21.3 \| + \| 28226756 \| 28234297 \| provirus \| 1 \| W \| Rhesus \| 27,93 \| \| 21q22.2 \| - \| 41111191 \| 41118353 \| pseudogene \| 1 \| W \| Gibbon \| 27,40 \| \| 22q12.3 \| - \| 34345530 \| 34345988 \| pseudogene \| 1 \| - \| Gibbon \| 38,21 \| \| Xp22.31 \| - \| 7617210 \| 7622109 \| pseudogene \| 1 \| W \| Rhesus \| 28,65 \| \| Xp11.3 \| + \| 46046300 \| 46049013 \| pseudogene \| 2 \| - \| Rhesus \| 36,42 \| \| Xp11.21 \| - \| 57424992 \| 57432088 \| pseudogene \| 1 \| W \| Rhesus \| 39,21 \| \| Xq12 \| - \| 65517016 \| 65519281 \| pseudogene \| 1 \| - \| Rhesus \| 33,82 \| \| Xq13.3 \| - \| 75118132 \| 75124909 \| provirus \| 1 \| W \| Gibbon \| 25,72 \| \| Xq21.1a \| - \| 77602103 \| 77606013 \| pseudogene \| 1 \| W \| Gibbon \| 43,79 \| \| Xq21.1b \| + \| 79211390 \| 79218603 \| pseudogene \| 1 \| R \| Orangutan \| 16,68 \| \| Xq22.3a \| + \| 105244784 \| 105248113 \| pseudogene \| 1 \| - \| Rhesus \| 25,84 \| \| Xq22.3b \| - \| 106295361 \| 106298094 \| pseudogene \| 1 \| - \| Rhesus \| 18,64 \| \| Xq23 \| - \| 115,871,924 \| 115,875,415 \| pseudogene \| 1 \| - \| Gibbon \| 22,36 \| \| Xq26.2 \| - \| 131063277 \| 131067083 \| undefined \| - \| - \| Rhesus \| - \| \| Xq27.1 \| + \| 139159755 \| 139162980 \| pseudogene \| 1 \| - \| Rhesus \| 23,46 \| \| Yp11.2 \| - \| 7754065 \| 7760683 \| provirus \| 2B \| ? \| Chimpanzee \| 72,52 \| \| Yq11.222 \| + \| 21241822 \| 21249383 \| provirus \| 1 \| W \| Chimpanzee \| 55,06 \| |
| --- | --- | --- | --- | --- | --- | --- | --- | --- | --- | --- | --- | --- | --- | --- | --- | --- | --- | --- | --- | --- | --- | --- | --- | --- | --- | --- | --- | --- | --- | --- | --- | --- | --- | --- | --- | --- | --- | --- | --- | --- | --- | --- | --- | --- | --- | --- | --- | --- | --- | --- | --- | --- | --- | --- | --- | --- | --- | --- | --- | --- | --- | --- | --- | --- | --- | --- | --- | --- | --- | --- | --- | --- | --- | --- | --- | --- | --- | --- | --- | --- | --- | --- | --- | --- | --- | --- | --- | --- | --- | --- | --- | --- | --- | --- | --- | --- | --- | --- | --- | --- | --- | --- | --- | --- | --- | --- | --- | --- | --- | --- | --- | --- | --- | --- | --- | --- | --- | --- | --- | --- | --- | --- | --- | --- | --- | --- | --- | --- | --- | --- | --- | --- | --- | --- | --- | --- | --- | --- | --- | --- | --- | --- | --- | --- | --- | --- | --- | --- | --- | --- | --- | --- | --- | --- | --- | --- | --- | --- | --- | --- | --- | --- | --- | --- | --- | --- | --- | --- | --- | --- | --- | --- | --- | --- | --- | --- | --- | --- | --- | --- | --- | --- | --- | --- | --- | --- | --- | --- | --- | --- | --- | --- | --- | --- | --- | --- | --- | --- | --- | --- | --- | --- | --- | --- | --- | --- | --- | --- | --- | --- | --- | --- | --- | --- | --- | --- | --- | --- | --- | --- | --- | --- | --- | --- | --- | --- | --- | --- | --- | --- | --- | --- | --- | --- | --- | --- | --- | --- | --- | --- | --- | --- | --- | --- | --- | --- | --- | --- | --- | --- | --- | --- | --- | --- | --- | --- | --- | --- | --- | --- | --- | --- | --- | --- | --- | --- | --- | --- | --- | --- | --- | --- | --- | --- | --- | --- | --- | --- | --- | --- | --- | --- | --- | --- | --- | --- | --- | --- | --- | --- | --- | --- | --- | --- | --- | --- | --- | --- | --- | --- | --- | --- | --- | --- | --- | --- | --- | --- | --- | --- | --- | --- | --- | --- | --- | --- | --- | --- | --- | --- | --- | --- | --- | --- | --- | --- | --- | --- | --- | --- | --- | --- | --- | --- | --- | --- | --- | --- | --- | --- | --- | --- | --- | --- | --- | --- | --- | --- | --- | --- | --- | --- | --- | --- | --- | --- | --- | --- | --- | --- | --- | --- | --- | --- | --- | --- | --- | --- | --- | --- | --- | --- | --- | --- | --- | --- | --- | --- | --- | --- | --- | --- | --- | --- | --- | --- | --- | --- | --- | --- | --- | --- | --- | --- | --- | --- | --- | --- | --- | --- | --- | --- | --- | --- | --- | --- | --- | --- | --- | --- | --- | --- | --- | --- | --- | --- | --- | --- | --- | --- | --- | --- | --- | --- | --- | --- | --- | --- | --- | --- | --- | --- | --- | --- | --- | --- | --- | --- | --- | --- | --- | --- | --- | --- | --- | --- | --- | --- | --- | --- | --- | --- | --- | --- | --- | --- | --- | --- | --- | --- | --- | --- | --- | --- | --- | --- | --- | --- | --- | --- | --- | --- | --- | --- | --- | --- | --- | --- | --- | --- | --- | --- | --- | --- | --- | --- | --- | --- | --- | --- | --- | --- | --- | --- | --- | --- | --- | --- | --- | --- | --- | --- | --- | --- | --- | --- | --- | --- | --- | --- | --- | --- | --- | --- | --- | --- | --- | --- | --- | --- | --- | --- | --- | --- | --- | --- | --- | --- | --- | --- | --- | --- | --- | --- | --- | --- | --- | --- | --- | --- | --- | --- | --- | --- | --- | --- | --- | --- | --- | --- | --- | --- | --- | --- | --- | --- | --- | --- | --- | --- | --- | --- | --- | --- | --- | --- | --- | --- | --- | --- | --- | --- | --- | --- | --- | --- | --- | --- | --- | --- | --- | --- | --- | --- | --- | --- | --- | --- | --- | --- | --- | --- | --- | --- | --- | --- | --- | --- | --- | --- | --- | --- | --- | --- | --- | --- | --- | --- | --- | --- | --- | --- | --- | --- | --- | --- | --- | --- | --- | --- | --- | --- | --- | --- | --- | --- | --- | --- | --- | --- | --- | --- | --- | --- | --- | --- | --- | --- | --- | --- | --- | --- | --- | --- | --- | --- | --- | --- | --- | --- | --- | --- | --- | --- | --- | --- | --- | --- | --- | --- | --- | --- | --- | --- | --- | --- | --- | --- | --- | --- | --- | --- | --- | --- | --- | --- | --- | --- | --- | --- | --- | --- | --- | --- | --- | --- | --- | --- | --- | --- | --- | --- | --- | --- | --- | --- | --- | --- | --- | --- | --- | --- | --- | --- | --- | --- | --- | --- | --- | --- | --- | --- | --- | --- | --- | --- | --- | --- | --- | --- | --- | --- | --- | --- | --- | --- | --- | --- | --- | --- | --- | --- | --- | --- | --- | --- | --- | --- | --- | --- | --- | --- | --- | --- | --- | --- | --- | --- | --- | --- | --- | --- | --- | --- | --- | --- | --- | --- | --- | --- | --- | --- | --- | --- | --- | --- | --- | --- | --- | --- | --- | --- | --- | --- | --- | --- | --- | --- | --- | --- | --- | --- | --- | --- | --- | --- | --- | --- | --- | --- | --- | --- | --- | --- | --- | --- | --- | --- | --- | --- | --- | --- | --- | --- | --- | --- | --- | --- | --- | --- | --- | --- | --- | --- | --- | --- | --- | --- | --- | --- | --- | --- | --- | --- | --- | --- | --- | --- | --- | --- | --- | --- | --- | --- | --- | --- | --- | --- | --- | --- | --- | --- | --- | --- | --- | --- | --- | --- | --- | --- | --- | --- | --- | --- | --- | --- | --- | --- | --- | --- | --- | --- | --- | --- | --- | --- | --- | --- | --- | --- | --- | --- | --- | --- | --- | --- | --- | --- | --- | --- | --- | --- | --- | --- | --- | --- | --- | --- | --- | --- | --- | --- | --- | --- | --- | --- | --- | --- | --- | --- | --- | --- | --- | --- | --- | --- | --- | --- | --- | --- | --- | --- | --- | --- | --- | --- | --- | --- | --- | --- | --- | --- | --- | --- | --- | --- | --- | --- | --- | --- | --- | --- | --- | --- | --- | --- | --- | --- | --- | --- | --- | --- | --- | --- | --- | --- | --- | --- | --- | --- | --- | --- | --- | --- | --- | --- | --- | --- | --- | --- | --- | --- | --- | --- | --- | --- | --- | --- | --- | --- | --- | --- | --- | --- | --- | --- | --- | --- | --- | --- | --- | --- | --- | --- | --- | --- | --- | --- | --- | --- | --- | --- | --- | --- | --- | --- | --- | --- | --- | --- | --- | --- | --- | --- | --- | --- | --- | --- | --- | --- | --- | --- | --- | --- | --- | --- | --- | --- | --- | --- | --- | --- | --- | --- | --- | --- | --- | --- | --- | --- | --- | --- | --- | --- | --- | --- | --- | --- | --- | --- | --- | --- | --- | --- | --- | --- | --- | --- | --- | --- | --- | --- | --- | --- | --- | --- | --- | --- | --- | --- | --- | --- | --- | --- | --- | --- | --- | --- | --- | --- | --- | --- | --- | --- | --- | --- | --- | --- | --- | --- | --- | --- | --- | --- | --- | --- | --- | --- | --- | --- | --- | --- | --- | --- | --- | --- | --- | --- | --- | --- | --- | --- | --- | --- | --- | --- | --- | --- | --- | --- | --- | --- | --- | --- | --- | --- | --- | --- | --- | --- | --- | --- | --- | --- | --- | --- | --- | --- | --- | --- | --- | --- | --- | --- | --- | --- | --- | --- | --- | --- | --- | --- | --- | --- | --- | --- | --- | --- | --- | --- | --- | --- | --- | --- | --- | --- | --- | --- | --- | --- | --- | --- | --- | --- | --- | --- | --- | --- | --- | --- | --- | --- | --- | --- | --- | --- | --- | --- | --- | --- | --- | --- | --- | --- | --- | --- | --- | --- | --- | --- | --- | --- | --- | --- | --- | --- | --- | --- | --- | --- | --- | --- | --- | --- | --- | --- | --- | --- | --- | --- | --- | --- | --- | --- | --- | --- | --- | --- | --- | --- | --- | --- | --- | --- | --- | --- | --- | --- | --- | --- | --- | --- | --- | --- | --- | --- | --- | --- | --- | --- | --- | --- | --- | --- | --- | --- | --- | --- | --- | --- | --- | --- | --- | --- | --- | --- | --- | --- | --- | --- | --- | --- | --- | --- | --- | --- | --- | --- | --- | --- | --- | --- | --- | --- | --- | --- | --- | --- | --- | --- | --- | --- | --- | --- | --- | --- | --- | --- | --- | --- | --- | --- | --- | --- | --- | --- | --- | --- | --- | --- | --- | --- | --- | --- | --- | --- | --- | --- | --- | --- | --- | --- | --- | --- | --- | --- | --- | --- | --- | --- | --- | --- | --- | --- | --- | --- | --- | --- | --- | --- | --- | --- | --- | --- | --- | --- | --- | --- | --- | --- | --- | --- | --- | --- | --- | --- | --- | --- | --- | --- | --- | --- | --- | --- | --- | --- | --- | --- | --- | --- | --- | --- | --- | --- | --- | --- | --- | --- | --- | --- | --- | --- | --- | --- | --- | --- | --- | --- | --- | --- | --- | --- | --- | --- | --- | --- | --- | --- | --- | --- | --- | --- | --- | --- | --- | --- | --- | --- | --- | --- | --- | --- | --- | --- | --- | --- | --- | --- | --- | --- | --- | --- | --- | --- | --- | --- | --- | --- | --- | --- | --- | --- | --- | --- | --- | --- | --- | --- | --- | --- | --- | --- | --- | --- | --- | --- | --- | --- | --- | --- | --- | --- | --- | --- | --- | --- | --- | --- | --- | --- | --- | --- | --- | --- | --- | --- | --- | --- | --- | --- | --- | --- | --- | --- | --- | --- | --- | --- | --- | --- | --- | --- | --- | --- | --- | --- | --- | --- | --- | --- | --- | --- | --- | --- | --- | --- | --- | --- | --- | --- | --- | --- | --- | --- | --- | --- | --- | --- | --- | --- | --- | --- | --- | --- | --- | --- | --- | --- | --- | --- | --- | --- | --- | --- | --- | --- | --- | --- | --- | --- | --- | --- | --- | --- | --- | --- | --- | --- | --- | --- | --- | --- | --- | --- | --- | --- | --- | --- | --- | --- | --- | --- | --- | --- | --- | --- | --- | --- | --- | --- | --- | --- | --- | --- | --- | --- | --- | --- | --- | --- | --- | --- | --- | --- | --- | --- | --- | --- | --- | --- | --- | --- | --- | --- | --- | --- | --- | --- | --- | --- | --- | --- | --- | --- | --- | --- | --- | --- | --- | --- | --- | --- | --- | --- | --- | --- | --- | --- | --- | --- | --- | --- | --- | --- | --- | --- | --- | --- | --- | --- | --- | --- | --- | --- | --- | --- | --- | --- | --- | --- | --- | --- | --- | --- | --- | --- | --- | --- | --- | --- | --- | --- | --- | --- | --- | --- | --- | --- | --- | --- | --- | --- | --- | --- | --- | --- | --- | --- | --- | --- | --- | --- | --- | --- | --- | --- | --- | --- | --- | --- | --- | --- | --- | --- | --- | --- | --- | --- | --- | --- | --- | --- | --- | --- | --- | --- | --- | --- | --- | --- | --- | --- | --- | --- | --- | --- | --- | --- | --- | --- | --- | --- | --- | --- | --- | --- | --- | --- | --- | --- | --- | --- | --- | --- | --- | --- | --- | --- | --- | --- | --- | --- | --- | --- | --- | --- | --- | --- | --- | --- | --- | --- | --- | --- | --- | --- | --- | --- | --- | --- | --- | --- | --- | --- | --- | --- | --- | --- | --- | --- | --- | --- | --- | --- | --- | --- | --- | --- | --- | --- | --- | --- | --- | --- | --- | --- | --- | --- | --- | --- | --- | --- | --- | --- | --- | --- | --- | --- | --- | --- | --- | --- | --- | --- | --- | --- | --- | --- | --- | --- | --- | --- | --- | --- | --- | --- | --- | --- | --- | --- | --- | --- | --- | --- | --- | --- | --- | --- | --- | --- | --- | --- | --- | --- | --- | --- | --- | --- | --- | --- | --- | --- | --- | --- | --- | --- | --- | --- | --- | --- | --- | --- | --- | --- | --- | --- | --- | --- | --- | --- | --- | --- | --- | --- | --- | --- | --- | --- | --- | --- | --- | --- | --- | --- | --- | --- | --- | --- | --- | --- | --- | --- | --- | --- | --- | --- | --- | --- | --- | --- | --- | --- | --- | --- | --- | --- | --- | --- | --- | --- | --- | --- | --- | --- | --- | --- | --- | --- | --- | --- | --- | --- | --- | --- | --- | --- | --- | --- | --- | --- | --- | --- | --- | --- | --- | --- | --- | --- | --- | --- | --- | --- | --- | --- | --- | --- | --- | --- | --- | --- | --- | --- | --- | --- | --- | --- | --- | --- | --- | --- | --- | --- | --- | --- | --- | --- |

^a^Classification based on phylogenetic analysis of 5’ and 3’ LTRs and gag and pol genes, supported by the identification of key mutations with respect to the epBase Update generated reference LTR17-HERV17-LTR17

^b^Oldest common ancestor, refers to most distant species that shares the sequence. Identified by RepeatMasker HERV17 annotations after BLAT searching for the primates orthologous locations in the Chimpanzee, Gorilla, Orangutan, Gibbon, Rhesus and Marmoset available genomes on the UCSC Genome Browser

^c^ estimated time of integration, in million years

^d^ Impossible to define due to the lack of the retroviral portion involved in classification

^e^ Impossible to unambiguously assign

*Sequence found as solitary LTR
