# Supplementary material for: Contribution of type W human endogenous retroviruses to the human genome: characterization of HERV-W proviral insertions and processed pseudogenes
Source: Retrovirology. 2016 Sep 9;13(1):67. doi: 10.1186/s12977-016-0301-x (PMC5016936; doi:10.1186/s12977-016-0301-x)
Supplement: Supplementary file 8 — 10.1186/s12977-016-0301-x HERV-W proposed consensus in Fasta format. HERV-W group general proviral consensus sequence (HERVW_PVcons) and HERV-W subgroups 1 and 2 consensus sequences (HERVW_SG1cons and HERVW_SG2cons, respectively) generated starting from our HERV-W sequences dataset. [file 12977_2016_301_MOESM8_ESM.docx]

Additional file S1: HERV-W proposed consensus

(LTRs are reported in bold)

>HERVW_PVcons

**tgagagacaggactagctggatttcctaggccgactaagaatccctaagcctagctgggaaggtgaccgcatccacctttaaacacggggcttgcaacttagctcacacccgaccaatcaggtagtaaagagagctcactaaaatgctaattaggcaaaaacaggaggtaaagaaatagccaatcatctatygcctgagagcacagsgggagggacaatgatcgggatataaacccaggcattcgagccggcaatggcaaccccctttgggtcccctcccwttgtatgggagctctgttttcactctattaaatcttgcaactgcactcttctggtccgtgtttgttacggctcgagctgagctttcgctcgccgtccaccactgctgtttgccgccgtcgcagacccgccrctgacttccayccctccggatccggcagggtgtccgctgtgctcctgatccagcgaggcrcccattgccrctcccgatygggctaaaggcttgccattgttcctgcayggctaagtgcctgggttcrtcctaatcgagctgaacactagtcactgggttccacggttctcttccrtgacccacggcttctaatagagctataacactcaccgcatggcccaagattccattccttggaatccgtgaggccaagaaccccaggtcagagaacaagaggcttgccrccatcttggaagcggccyrccaccatcttggaagyggcccgccaccatcttgggagctctaagaacaaggacccccccggtaacattttggcgaccatgaagggacctccaaagcggtgagta**atattggaccactttcgcttgctattctgtcctatccttccttagaattggaggaaaataccgggcacctgtcggccagttaaaaacgattagcgtggccgccggacttaagactcaggtgtgaggctatctggggaagggctttctaacaacccccaacccttctgggttgggagcgttggtctgcctggaaccagcttccactttcacaattttcttggggaagccgagggctgactagaggcagaaagctgtcgtcccgaactcccggcattagccggttgagatcatggcgcagccagaagtctctactcaacagtcgcccatgcgtgcgcccctacctttccttctgacccatacctcctgggtcccgaccatgactttcttgaaagtgtagccccaaaattctccttacctctgaatctacttcctctgatccctgcctcctaggtactaatggttcagactttcatttcctctcccaagtattagagcaagttgtatctccaaagggatctaaggaagctctacgctgtgtccttaggcacctaggctatgaacccagggagtcttgtccctggtgtccctcccaatttaggcatacagctctcgacatgggcagttatgtgggacccgttccccaccacccttgccagggccccaagtttgtaaatggctaggaggattgctctcccattgtgtaagatgctctcctcccccaatttctacccatcttacccccctgcaatacaatctccaagccttggctccttggccagggccttagaactgatgacccagtacttaaatggctggaactgggtctacaacaacataatagatcaggatgaaagcgaattgagtaaattaaagggaggcgcatattcctatagtggcaaatgggggcaacgagcaaacgtccttccactgtgttcccaaaatccatctacaaagagagagagaggaaagagaagagagagagagagagagagagagagagagagagagagagagagagagagagagagagagaggagagaggagagagagagagagagagagaagagagagagaagaagagacaaagagggagtcaaagagagaaagaaagagaaagatagaagtagaaaaaaaaaaacagtgtgccctattcctttaaaagccagggtaaatttaaaacctataattgataattgaaggtcttctccgtgaccctataacactccaatactaccttgttgtcagtgtaaacaagggcgtagcctgaaaacactgagaccactgacaacccgtagccttcctatcaaaaatccttaacccagtaacccacggatggcccaaatgcattcaatctgtagcagcaactgctttgctaacagaagaaagtagaaaagtaacttttagaggaaacctcattgtgagcacacctcaccagttcagaattattctaagtcaaaaaagcaaaaaggtagcttactaactcaaaaatcttaaagtatggggctattctgttagaaaaaggtaatttaacactaaccactgaaaattcccttaacccagcagatttcctaacaggggatttaaatcttaattaccatacaaaggtccgaccagacctaggaggaactcccttcaggacaggacgatagatggttcctcccaggtgattgaggaaaaaaccacaatgggtattcagtaattgatagggagactcttgtggaagcagagttaggaaaattgcctaataattggtctgctcaaacgtgcgagctgtttgcactcagccaagccttaaagtacttacagaatcaaaaaaactctatctcaatcctgactcaaaaggttacctacaccctctctgaaatgaatttgcataagaactgttgtttatgggaatgcatcttgatggggcagctgggttgttatgaaatactcaggaacccagcccagctctaggactcacccctgagcacaaaggcaatgttgggcacgctggtaaaggaccactagaatccagcagcccggacccctttctttgtggtcaagaaaggcgggaaaacaggtgcaggactgctacatcggtgagcgtaactaatccgataagcagaggtccatgggtggttacgcaccctggaaaggaataagcattaggaccatagaggacgctctaggactaatgctcatcggaaaatgactaggggtgctggcatccctatgttcttttttcagatgggaaacgttccccccaaggcaaaaacgcccctaagatgtattctggagaattgggaccaatttgaccctcagacgctaagaaagaaatgacttatattcttctgcagtaccgcctggccacgatatcctcttcaagggggagaaacctggcctcctgagggaagtataaattataacaccatcttacagctagacctcttttgtagaaaagaaggcaaatggagtgaagtgccatatgtacaaactttcttttcattaagagacaactcgcaattatgtaaaaagtgtgatttatgccctacaggaagccctcagagtctacctccctaccccagcgtccccccgactccttccccaactaataaggaccccccttcaacccaaacggtccaaaaggagatagacaaaggggtaaacaatgaaccaaagagtgccaatattccccgattatgccccctccaagcagtgggaggaggagaattcggcccagtcagagtgcatgtacctttttccctctcagacttgaagcaaattaaaatagacctaggtaaattctcagataaccctgatggctatattgatgttttacaagggttaggacaatcctttgatctgacatggagagatataatgttactgctagatcagacactaaccccaaatgagagaagtgccgccataactgcagcccgagagtttggcgatctctggtatctcagtcaggtcaatgataggatgacaacagaggaaagagaacaattccccacaggccagcaggcagttcccagtgtagaccctcactgggacacagaatcagaacatggagattggtgccgcagacatttgctaacttgcgtgctagaaggactaaggaaaactaggaagaagcctatgaattattcaatgatgtccactataacacagggaaaggaagaaaatcctactgcctttctggagagactaagggaggcattgaggaagcatacctctctgtcacctgactctattgaaggccaactaatcttaaaggataagtttatcactcagtcagctgcagacattagaaaaaaacttcaaaagtccgccttaggcccggagcaaaacttagaaaccctattgaacttggcaacctcggttttttataatagagatcaggaggagcaggcggaacgggacaaacgggataaaaaaaaggccaccgctttagtcatggccctcaggcaagcggactttggaggctctggaaaagggaaaggctgggcaaatcgaatgcctaatagggcttgcttccagtgcggtctacaaggacactttaaaaaagattgtccaaatagaaataagccgccccctcgtccatgccccttatgtcaagggaatcactggaaggcccactgccccaggggatgaaggtcctctgagtcagaagccactaaccagatgatccagcagcaggactgagggtgcccggggcaagcgccagcccatgccatcaccctcacagagccccgggtatgcttgaccattgagggccaggaggttaactgtctcctggacactggcgcggccttctcagtcttactctcctgtcccggacaactgtcctccagatctgtcactatccgaggggtcctaggacagccagtcactagatacttctcccagccactaagttgtgactggggaactttactcttttcacatgcttttctaattatgcctgaaagccccactcccttgttagggagagacattctagcaaaagcaggggccattatacacctgaacataggagaaggaacacccgtttgttgtcccctgcttgaggaaggaattaatcctgaagtctgggcaacagaaggacaatatggatgagcaaagaatgcccatcctgttcaagttaaactaaaggattccacctcctttccctaccaaaggcagtacccccttagacccgaggcccaacaaggactccaaaagattgttaaggacctaaaagcccaaggcctagtaaaaccatgcaatagcccctgcaatactccaattttaggagtacagaaacccaacggacagtggaggttagtgcaagatctcaggattatcaatgaggccgttgtccctctatacccagctgtacctaacccttatactctgctttcccaaataccagaggaagcagagtggtttacagtcctggaccttaaggatgcctttttctgcatccctgtacatcctgactctcaattcttgtttgcctttgaagatccttcgaacccaacgtctcaactcacctggactgttttaccccaagggttcagggatagcccccatctatttggccaggcattagcccaagacttgagccagttctcatacctggacactcttgtccttcggtacgtggatgatttacttttagccacccgttcagaaaccttgtgccatcaagccacccaagcgctcttaaatttcctcgccacctgtggctacaaggtttccaaaccaaaggctcagctctgctcacagcaggttaaatacttagggctaaaattatccaaaggcaccagggccctcagtgaggaatgtatccagcctatactggcttatcctcatcccaaaaccctaaagcaactaagagggttccttggcataacaggcttctgccgaatatggattcccaggtacggcgaaatagccaggccattatatacactaattaaggaaactcagaaagccaatacccatttagtaagatggacacctgaagcagaagcggctttccaggccctaaagaaggccctaacccaagccccagtgttaagcttgccaacggggcaagacttttctttatatgtcacagaaaaaacaggaatagctctaggagtccttacacaggtccgagggacgagcttgcaacccgtggcatacctgagtaaggaaattgatgtagtggcaaagggttggcctcattgtttacgggtagtggcagcagtagcagtcttagtatctgaagcagttaaaataatacagggaagagatcttactgtgtggacatctcatgatgtgaacggcatactcactgctaaaggagacttgtggctgtcagacaaccatttacttaaatatcaggctctattacttgaagggccagtgctgcgactgcgcacttgtgcaactcttaacccagccacatttcttccagacaatgaagaaaagatagaacataactgtcaacaagtaattgctcaaacctatgccgctcgaggggaccttctagaggttcccttgactgatcccgacctcaacttgtatactgatggaagttcctttgtagaaaaaggacttcgaaaagcggggtatgcagtggtcagtgataatggaatacttgaaagtaatcccctcactccaggaactagtgctcagctggcagaactaatagccctcactcgggcactagaattaggagaaggaaaaagggtaaatatatatacagactctaagtatgcttacctagtcctccatgcccacgcagcaatatggagagaaagggaattcctaacttccgagggaacacctatcaaacatcaggaagccattaggagattattattggctgtacagaaacctaaagaggtggcagtcttacactgccggggtcatcagaaaggaaaggaaagggaaatagaagggaaccgccaagcggatattgaagccaaaagagccgcaaggcaggaccctccattagaaatgcttatagaaggacccctagtatggggtaatcccctccaggaaaccaagccccagtactcagcagaagaaatagaatggggaacctcacgaggacatagtttcctcccctcaggatggctagccaccgaagaaggaaaaatacttttgcctgcagctaaccaatggaaattacttaaaacccttcaccaaacctttcacttaggcattgatagcacccatcagatggccaaatcattatttactggaccaggccttttcaaaactatcaagcagatagtcagggcctgtgaagtgtgccaaagaaataatcccctgccttatcgccaagctccttcaggagaacaaagaacaggccattacccaggagaagactggcaactagattttacccacatgcccaaatctcagggatttcagtatctactagtctgggtagatactttcactggttgggcagaggccttcccttgtaggacagaaaaggcccaagaggtaataaaggcactaattcatgaaataattcccagattcggacttccccgaggcttacagagtgacaatggccccgctttcaaggctgcagtaacccagggagtatcccaggcgttaggcatacaatatcacttacactgcgcctggaggccacaatcctcaggaaaagtcgagaaaatgaatgaaacactcaaacgacatctaaaaaagctaacccaagaaacccacctcgcatggcctgctctgttgcctatagccttactaagaatccaaaactctccccaaaaagcaggacttagcccatacgaaatgctgtatggacggcccttcctaaccaatgaccttgtgcttgaccgagagatggccaacttagttgcagacatcacctccttagccaaatatcaacaagttcttaaaacattacagggaacctgtccccgagaggagggaaaggaattattccaccctggtgacatggtattagtcaagtcccttccctctaattccccatccctagatacatcctgggaaggaccctacccagtcattttatctaccccaaccgcggttaaagtggctggagtggagtcttggatacatcacactcgagtcaaaccctggatactgccaaaggaacccgaaaatccaggagacaacgctagctattcctgtgaacctctagaggatctgcgcctgctcttcaagcgacaaccgtgaggaaagtaactaaaatcgtaaatccccatggccctcccttatcatatttttctctttactgttctcttaccccctttcactctcactgcaccccctccatgccactgtactaccagtagctccccttaccaagagcttctatggagaatgcggcttcccggaaatattgatgccccatcgtataggagtttttctaaaggaaaccccaccttcaccgcccacacccatatgccccgcaactgctataactctgccactctttgcatgcatgcaaatactcattattggacagggaaaatgattaatcctagttgtcctggaggacttggagccactgtctgttggacttacttcacccataccggtatgtctgatgggggtggagttcaagatcaggcaagagaaaaacacgtaaaggaagtaatctcccaactgacccgggtacatagcacccctagcccctacaaaggactagatctctcaaaactacatgaaaccctccgtacccatactcacctggtaagcctatttaataccaccctcactgggctccatgaggtcttggcccaaaaccctactaactgttggatgtgcctccccctgcacttcaggccatacatttcaatccctgtacctgaacaatggaacaacttcagcacagaaataaacaccacttccattttagtaggacctcttgtttccaatctggaaataacccatacctcaaacctcacctgtgtaaaatttagcaatactatagacacaaccaactcccaatgcatcaggtgggtaactcctcccacacaaatagtctgcctaccctcaggaatattttttgtctgtggtacctcagcctatcattgtttgaatggctcttcagaatctatgtgcttcctctcattcttagtgccccctatgaccatctacactgaacaagatttatacagttatgtcgtacctaagccccgcaacaaaagagtacccattcttccttttgttatcggagcaggagtgctaggtggactaggtactggcattggcggtatcacaacctctactcagttctactacaaactatctcaagaactaaatggtgacatggaacgggtcgccgactccctggtcaccttgcaagatcaacttaactccctagcagcagtagtccttcaaaatcgaagagctttagacttgctaaccgctgaaagagggggaacctgtttatttttaggggaagaatgctgttattatgttaatcaatccggaatcgtcaccgagaaagttaaagaaattcgagatcgaatacaacgtagagcagaggagcttcgaaacactggaccctggggcctcctcagccaatggatgccctggattctccccttcttaggacctctagcagctataatattgttactcctctttggaccctgtatctttaacctccttgttaagtttgtctcttccagaatcgaagctgtaaaactacaaatcgttcttcaaatggagccccagatgcagtccatgactaagatctaccgcggacccctggaccggcctgctagcccatgctccgatgttaatgacatcgaaggcacccctcccgaggaaatctcaactgcacaacccctactacgccccaattcagcaggaagcagttagag**cggtcatcggccaacctccccaacagcacttgggttttcctgttgagaggggggactgagagacaggactagctggatttcctaggccgactaagaatccctaagcctagctgggaaggtgaccgcatccacctttaaacacggggcttgcaacttagctcacacccgaccaatcaggtagtaaagagagctcactaaaatgctaattaggcaaaaacaggaggtaaagaaatagccaatcatctatygcctgagagcacagsgggagggacaatgatcgggatataaacccaggcattcgagccggcaatggcaaccccctttgggtcccctcccwttgtatgggagctctgttttcactctattaaatcttgcaactgcactcttctggtccgtgtttgttacggctcgagctgagctttcgctcgccgtccaccactgctgtttgccgccgtcgcagacccgccrctgacttccayccctccggatccggcagggtgtccgctgtgctcctgatccagcgaggcrcccattgccrctcccgatygggctaaaggcttgccattgttcctgcayggctaagtgcctgggttcrtcctaatcgagctgaacactagtcactgggttccacggttctcttccrtgacccacggcttctaatagagctataacactcaccgcatggcccaagattccattccttggaatccgtgaggccaagaaccccaggtcagagaacaagaggcttgccrccatcttggaagcggccyrccaccatcttggaagyggcccgccaccatcttgggagctctaagaacaaggacccccccggtaaca**

>HERVW_SG1cons

**tgagagacaggactagctggatttcctaggccgactaagaatccctaagcctagctgggaaggtgaccgcatccacctttaaacacggggcttgcaacttagctcacacccgaccaatcaggtagtaaagagagctcactaaaatgctaattaggcaaaaacaggaggtaaagaaatagccaatcatctattgcctgagagcacagcgggagggacaatgatcgggatataaacccaggcattcgagccggcaacggcwacccyctttgggtcccctccctttgtatgggagctctgttttcactctattaaatcttgcaactgcactcttctggtccrtgtttgttacggctcgagctgagctttcgctcrccgtccaccactgctgtttgccgccgtcgcagacccgccrctgacttccayccctccggatccggcagggtgtccgctgtgctcctgatccagygaggcrcccattgccrctcccgatygggctaaaggcttgccattgttcctgcayggctaagtgcctgggttcrtcctaatcgagctgaacactagtcactgggttccacggttctcttccrtgacccacggcttctaatagagctataacactcaccgcatggcccaagattccattccttggaatccgtgaggccaagaaccccaggtcagagaacacgaggcttgccaccatcttggaagyggcccgccaccatcttgggagctctgggagcaaggaccccccggtaaca**ttttggcgaccacgaagggacctccaaagcggtgagtaatattggaccactttcgcttgctattctgtcctatccttccttagaattggaggaaaataccgggcacctgtcggccagttaaaaacgattagcrtggccgccggacttaagactcaggtgtgaggctrtctggggaagggctttctaacaacccccaacccttctgggttgggracgttggtctgcctggagccagcttccactttcaattttcttggggaagccgagggccgactagaggcagaaagctgtcgtcccgaactcccggcattagccggttgagatcatggcgcagccagaagtctctactcaacagtcgcccatgcgtgcgcccctacctttccttctgacccatacctcctgggtcccgaccaygactttcttgaaagtgtagccccaaaattctccttacctctgaatctacttcctctgatccctgcctcctaggtactaatggttcagactttcatttcctctagcaagttgtatctccaaagggatctaaggaagctctaygctgygtccttaggcayctaggctataaacccagggagtcttatccctggtgtccctcccratttaggtatacagctctcgacatgggcagttatgtgggacccgttccccaccacccttgccagggccccaagtttgtaaagggctaggagaaaagagagacagaggagagagagagagacggaggagagagagagagagacagagaggagagagagacaggagagagacagaagagagagagagacagagaggagagagagagagagagagagagagargagagagagagagagagagagagagagaaaagagaaaagtgtgccctattcctttaaaagccagggtaaatttaaaacctataattgataattgaaggtcttctccrtgaccctataacactccaatactaccttgttgtcagtgtaaacaagggcgtagcctgaaarcactgagaccactgacaacccgtagccttcctatcaaaaatccttaacccagtaacccrcggatggcccaaatgcattcaatctgtagcggcaactgctttgctaacagaagaaagtagaaaagtaacttttagaggaaacctcattgtgagcacacctcaccagttcagaattattctaagtcaaaaaagcaaaaaggtagcttactaactcaaaaatcttaaagtatggggctattctgttagaaaaaggtaatttaacacyaaccactgataattcccttaacccagcagatttcctaacaggggatttaaatcttaattaccatacaaaggtccgaccagacctaggaggaactcccttcaggacaggacgatagatggttcctcccaggtgattgaggaaaaaaccacaatgggtattcagtaattgatagggagactcttgtggaagcagagttagaaaaattgcctaataattggtctcctcaaacgtgcgagctgtttgcactcagccaagccttaaagtacttacagaatcaaaaaactatctcaatcctgactcaaaaggttacctacaccctctctgaaacgaatttgcataagaactgttgtttatgggaatgcatcttgatggggcagctgggttgttatgaaatactcaggaacccagcccagctctaggactcacccctgagcacaaaggcaatgttgggcacgctggtaaaggaccactagaatccagcagcccggacccctttctttgtggtcaagaaaggcgggaaaacaggtgcaggactgctacatcggtgagcgtaactaatccgataagcagaggtccatgggtggttacgcaccctggaaaggaataagcattaggaccatagaggacgctctaggactaatgctcatcggaaaatgactaggggtgctggcatccctatgttcttttttcagatgggaaacgttccccccaaggcaaaaacgcccctaagatgtattctggagaattgggaccaatttgaccctcagacgctaagaaagaaatgacttatattcttctgcagtaccgcctggccacgatatcctcttcaagggggagaaacctggcctcctgagggaagtataaattataacaccatcttacagctagacctcttttgtagaaaagaaggcaaatggagtgaagtgccatatgtacaaactttcttttcattaagagacaactcgcaattatgtaaaaagtgtgatttatgccctacaggaagccctcagagtctacctccctaccccagcrtccccccgactccttccccaactaataaggaccccccttcaacccaaacggtccaaaaggagatagacaaaggggtaaacaatgaaccaaagagtgccaatattccccgattatgccccctccaagcagtgggaggaggagaattcggcccagccagagtgcatgtacctttttctctctcagacttaaagcaaattaaaatagacctaggtaaattctcagataaccctgatggctatattgatgttttacaagggttaggacaatcctttgatctgacatggagagatataatgttactgctaaatcagacactaaccccaaatgagagaagtgccgccataactgcagcccgagagtttggcgatctctggtatctcagtcaggtcaatgataggatgacaacagaggaaagagaacgattccccacaggccagcaggcagttcccagtgtagaccctcactgggacacagaatcagaacatggagattggtgccgcagacatttgctaacttgcgtgctagaaggactaaggaaaactaggaagaagcctatgaattattcaatgatgtccactataacacagggaaaggaagaaaatcctactgcctttctggagagactaagggaggcattgaggaagcatacctctctgtcacctgactctattgaaggccaactaatcttaaaggataagtttatcactcagtcagctgcagacattagaaaaaaacttcaaaagtcygccttaggcccggagcaaaacttagaaaccctattgaacttggcaacctcggttttttataatagagatcaggaggagcaggcggaacgggacaaacgggataaaaaaaaggccaccgctttagtcatggccctcaggcaagcggactttggaggctctggaaaagggaaaagctgggcaaatcgaatgcctaatagggcttgcttccagtgcggtctacaaggacactttaaaaaagattgtccaagtagaaataagccgccccctcgtccatgccccttatgtcaagggaatcactggaaggcccactgccccaggggatgaaggtcctctgagtcagaagccactaaccagatgatccagcagcaggactgagggtgcccggggcaagcgccagcccatgccatcaccctcacagagccccgggtatgcttgaccattgagggccaggaggttaactgtctcctggacactggcgcggccttctcagtcttactctcctgtcccggacaactgtcctccagatctgtcactatccgaggggtcctaggacagccagtcactagatacttctcccagccactaagttgtgactggggaactttactcttttcacatgcttttctaattatgcctgaaagccccactcccttgttagggagagacattctagcaaaagcaggggccattatacacctgaacataggagaaggaacacccgtttgttgtcccctgcttgaggaaggaattaatcctgaagtctgggcaacagaaggacaatatggatgagcaaagaatgcccrtcctgttcaagttaaactaaaggattccgcctcctttccctaccaaaggcagtacccccttagacccgaggcccaacaaggactccaaaagattgttaaggacctaaaagcccaaggcctagtaaaaccatgcaatagcccctgcaatactccaattttaggagtacagaaacccaacggacagtggaggttagtgcaagatctcaggattatcaatgaggcygttgtccctctatacccagctgtacctaacccttatactctgctttcccaaataccagaggaagcagagtggtttacagtcctggaccttaaggatgcctttttctgcatccctgtacatcctgactctcaattcttgtttgcctttgaagatccttcgaacccaacgtctcaactcacctggactgttttaccccaagggttcagggatagcccccatctatttggccaggcattagcccaagacttgagccagttctcatacctggacactcttgtccttcggtacgtggatgatttacttttagccrcccgttcagaaaccttgtgccatcaagccacccaagcgctcttaaatttcctcgctacctgtggctacaaggtttccaaaccaaaggctcagctctgctcacagcaggttaaatacttagggctaaaattatccaaaggcaccagggccctcagtgaggaaygtatccagcctatactggcttatcctcatcccaaaaccctaaagcaactaagagggttccttggcataacaggyttctgccgaatatggattcccaggtacggcgaaatagccaggccattatatacactaattaaggaaactcagaaagccaatacccatttagtaagatggacacctgaagcagaagcggctttccaggccctaaagaaggccctaacccaagccccagtgttaagcttgccaacggggcaagacttttctttatatgtcacagaaaaaacaggaatagctctaggagtccttacacaggtccgagggacgagcttgcaacccgtggcatacctgagtaaggaaattgatgtagtggcaaagggttggcctcattgtttacgggtagtggcggcagtagcagtcttagtatctgaagcagttaaaataatacagggaagagatcttactgtgtggacatctcatgatgtgaayggcatactcactgctaaaggagacttgtggctgtcagacaaccgtttacttaaatatcaggctctattacttgaagggccagtgctgcgactgcgcacttgtgcaactcttaacccagccacatttcttccagacaatgaagaaaagatagaacataactgtcaacaagtaattgctcaaacctaygccactcgaggggaccttttagaggttcccttgactgatcccgacctcaacttgtatactgatggaagttcctttgtagaaaaaggacttcgaaaagcggggtatgcagtggtcagtgataatggaatacttgaaagtaatcccctcactccaggaactagtgctcagctggcagaactaatagccctcactcgggcactagaattaggagaaggaaaaagggtaaatatatatacagactctaagtatgcttacctagtcctccatgcccatgcagcaatatggagagaaagggaattcctaacttccgagggaacacctatcaaacatcaggaagccattaggagattattattggctgtacagaaacctaaagaggtggcagtcttacactgccggggtcatcagaaaggaaaggaaagggaaatagaagggaaccgccaagcggatattgaagccaaaagagccgcaaggcaggaccctccattagaaatgcttatagaaggacccctagtatggggtaatcccctccgggaaaccaagccccagtactcagcagaagaaatagaatggggaacctcacgaggacatagtttcctcccctcaggatggctagccaccgaagaaggaaaaatacttttgcctgcagctaaccaatggaaattacttaaaacccttcaccaaacctttcacttaggcattgatagcacccatcagatggccaaattattatttactggaccaggccttttcaaaactatcaagcagatagtcagggcctgtgaagtgtgccaaagaaataatcccctgccttatcgccaagctccttcaggagaacaaagaacaggccattacccaggagaagactggcaactagattttacccacatgcccaaatctcagggatttcagtatctactagtytgggtagatactttcactggttgggcagaggccttcccctgtaggacagaaaaggcccaagaggtaataaaggcactagttcatgaaataattcccagattcggacttccccgaggcttacagagtgacaatggccctgctttcaaggctacagtaacccagggagtatcccaggcgttaggtatacaatatcacttacactgcgcctggaggccacagtcctcagggaaggtcgagaaaatgaatgaaacactcaaaygacatctaaaaaagctaacccaggaaacccacctcgcatggcctgctctgttgcctatagccttactaagaatccgaaactctccccaaaaagcaggacttagcccatacgaaatgctgtatggacggcccttcctaaccaatgaccttgtgcttgaccgagagacggccaacttagttgcagacatcacctccttagccaaatatcaacaagttcttaaaacattacaaggagcctgtccccgagaagagggaaaggaactattccaccctggtgacatggtattagtcaagtcccttccctctaattccccatccctagatacatcctgggaaggaccctacccagtcattttatctaccccaaccgcggttaaagtggctggagtggagtcttggatacatcacactcgagtcaaaccctggatactgccaaaggaacccgaaaatccaggagacaacgctagctattcctgtgaacctctagaggatctgcgcctgctcttcaagcracaaccgtgaggaaagtaactaaaatcrtaaatccccatggccctcccttatcatatttttctctttactgttctcttaccccctttcactctcactgcaccccctccatgccactgtacraccagtagctccccttaccaagagtttctatggagaatgcggcttcccggaaatattgatgccccatcgtataggagtttatctaagggaaaccccaccttcactgcccacacccatatgccccacaactgctataactctgccactctttgcatgcatgcaaatactcattattggacagggaaaatgattaatcctagttgtcctggaggacttggagccactgtctgttggacttacttcacccataccggtatgtctgatgggggtggagttcaagatcaggcaagagaaaaacacgtaaaggaagtaatctcccaactgacccgggtacatagcacccctagcccctacaaaggactagatctctcaaaactacatgaaaccctccgtacccatactcgcctggtaagcctatttaataccaccctcactgggctccatgaggtctcggcccaaaaccctactaactgttggatgtgcctccccctgcactgcaggccatacatttcaatccctgtacctgaacaatggaacaacttcagcacagaaataaacaccacttccgttttagtaggacctcttgtttccaatctggaaataacccatacctcaaacctcacctgtgtaaaatttagcaatactatagacacaaccaactcccaatgcatcaggtgggtaactcctcccacacgaatagtctgcctaccctcaggaatattttttgtctgtggtacctcagcctatcgttgtttgaatggctcttcagaatctatgtgcttcctctcattcttagtgccccctatgaccatctacactgaacaagatttatacaattatgtcgtacctaagccccgcaacaaaagagtacccattcttccttttgttatcggagcaggagtgctaggtggactaggtactggcattggcggtatcacaacctctactcagttctactacaaactatctcaagaactaaatggtgacatggaacgggtcgccgactccctggtcaccttgcaagatcaacttaactccctagcagcagtagtccttcaaaatcgaagagctttagacttgctaaccgccgaaagagggggaacctgtttatttttaggggaagaatgctgttattatgttaatcaatccggaatcgtcaccgagaaagttaaagaaattcgagatcgaatacaacgtagagcagaggagcttcaaaacactggaccctggggcctcctcagccaatggatgccctggattctccccttcttaggacctctagcagctataatattgttactcctctttggaccctgtatctttaacctccttgttaagtttgtctcttccagaatcgaagctgtaaaactacaaatcgttcttcaaatggagccccagatgcagtccatgactaagatctaccgcggacccctggaccggcctgctagcccatgctccgatgttaatgacatcgaaggcacccctcccgaggaaatctcaactgcacaacccctactatgccccaattcagcaggaagcagttagagcggtcatcggccaacctccccaacagcacttgggttttcctgttgagaggggggac**tgagagacaggactagctggatttcctaggccgactaagaatccctaagcctagctgggaaggtgaccgcatccacctttaaacacggggcttgcaacttagctcacacccgaccaatcaggtagtaaagagagctcactaaaatgctaattaggcaaaaacaggaggtaaagaaatagccaatcatctattgcctgagagcacagcgggagggacaatgatcgggatataaacccaggcattcgagccggcaacggcwacccyctttgggtcccctccctttgtatgggagctctgttttcactctattaaatcttgcaactgcactcttctggtccrtgtttgttacggctcgagctgagctttcgctcrccgtccaccactgctgtttgccgccgtcgcagacccgccrctgacttccayccctccggatccggcagggtgtccgctgtgctcctgatccagygaggcrcccattgccrctcccgatygggctaaaggcttgccattgttcctgcayggctaagtgcctgggttcrtcctaatcgagctgaacactagtcactgggttccacggttctcttccrtgacccacggcttctaatagagctataacactcaccgcatggcccaagattccattccttggaatccgtgaggccaagaaccccaggtcagagaacacgaggcttgccaccatcttggaagyggcccgccaccatcttgggagctctgggagcaaggaccccccggtaaca**

>HERVW_SG2cons

**tgagagacaggactagctggatttcctaggccgactaagaattcctaagcctagctggggaaggtgacyrcacccacctttaaacayggggcttgtaactcagctcacacccgaccaatcaggtagtaaagagrgctcactaaaatacmaattaggctaaaagcaggaggtaaagaaatagtcaaatcatatatcrcctgagagcacagggggagggacaatgatygggatataaacccaggcattcgagcmgggagtggcaaccccctttgggtcccctcccattgtatgggagctctgttttcactctattaaatcttgcaactgcacactcttctggtccgtgtttgttmcggctcragctgagctttcgctcrccgtccaccactgctgtttgccgccgtcgcagacccgccactgacttccacccctcyggatccggcagggtgtccrctgygctyctgatccagygaggcgcccattgccrctccygatcgggctaraggctcrccattgttcctgcatggctaagtgcccgggttcrtcctaatcgagctgaacactagtcgctgggttccacggttctcttccrtgacccacggcttctaatagagctataacactcaccgcatggcccaaggttccattccttggaatccgtgaggccaagaaccccaggtcagagaacaaaaggcttgctgccatcttggragcggcccgccaccatcttgggagctctaagaacaaagacccmccmgtaaca**tttggtggccccatahagggattctccaaagcggtgagtaatattggaccactttcgcttgctattctgtcctatccttccttagaattggaggaaaataccgggcacctgtcrgccagttaaaaacgattagcgtggcygccggacttaagactcaggtgtgaggctttctgggaaaaggctttctaacaacccccaacccttctgggttgggagcattggtctgcctggaaccagcttccgctttcacaattttcctggggaagccgagggccgactagaggcagaaagctgtcgtcccgaactcccggcattggccggttgagatcatggcrcagccagaagtctctactcaacagtcgcccatgcgtgcrcccctacctytccttctgacccatacctcctgggtccygaccatgactttcttgaaagtgtagccccaaaattctccttacctctgaatctacttcctcygatccctgcctcctaggtactaatgcttcagactttcacttcctctcccaagtattagagcaagttgtatctccaaagggatctaaggaagctctaygctgtgtccttaggcacctaggctatgaacccagggagtcttgtccctggtgtccctcccaatttaggtatacagctctcgacatgggcagttatgtgggacccrttccccaccacccttgccagccccaagtttgtaaatggctaggaggattgctctcccattgtgtaagatgctctcctcccccaatttctacccagcttacccctctgcaatacaatctccaagccttggctccttggccagggccttagaactgatracccagtactttaacaactggaactgggtctacracaacataatagatcaggatgaaagcgaattgagtaaattaaagggaggcacatattcctatagtggcaaatgggggcaactagcaaacgtccttccactgtgttcccaaaatccatctacaaagagagagagaggaaagaaagaaaagagagagagagaagagaaagagagaagagagaaagagagagaaaaaagaaagagagaggaaaaagaaagaaagagaaagaragagagagagagagagagaaagagaaagatagaagtagtaaagaaaaaacagtgtgccctattcctttaaaagccagggtaaatttaaaacctataattgataattgaaggtcttctccgtgaccctataacactccaatactaccttgttgtcagtgtaaacaagggcgtagcctgaaaacactgagaccactgacaacccgtagccttcctatcaaaaatccttaacccagtaacccrcggatggcccaaatgcattcaatctgtagcggcaactgctttgctaacagaagaaagtagaaaaataacttttagaggaaacctcattgtgagcacacctcaccagttcagaactatcctaagtcaaaaaagcaaaaaggtagcttactaactcaaaaatcttaaagtatggggctattctgttagaaaaaggtgatttaacattaaccactgaaaattcccttaacccagcagatttcctaacaggggatttaaatcttaattaccatacaaaggtccgaccagacctaggaggaactcccttcaggacaggacgatagatggttcctcccaggtgattgagaaaaaaaaccacaatgggtattcagtaattgatagggaaactcttgtrgaagcagagttaggaaaattgcctaataattggtctgctcaaacgtgcgagctgtttgcactcagccaagccttaaagtacttacagaataaaaactctatctcaatcctgactcaaaaggttacctacaccctctctgaaatgaatttgcataagaactgttgtttatgggaatgcatcttgatggggcagctgggttgttatgaaatactcaggaacccagcccagctctagractcacccctgagcacaaaggcaatgttgggcacgctggtaaaggaccactagaatccagcagccyggacccctttctttgtggtcaagaaaggcrggaaaacaggtgcaggactgctacatcggtgagcgtaactaatccgataagcagaggtccatgggtggttacgcaccctggaaaggaataagcattaggaccatagaggacgctctaggactaatgctcatcggaaaatgactaggggtgctggcatccctatgttcttttttcagatgggaaacrttccccccaaggcaaaaacgcccctaagatgtattctggagaattgggaccaatttgaccctcagacgctaagaaagaaacgacttatattcttctgcagtaccgcctggccacgatatcctcttcaagggggagaaacctggcctcctgagggaagtataaattataacaccatcttacagctagacctcttttgtagaaaagaaggcaaatggagtgaagtgccatatgtacaaactttcttttcattaagagacaactcgcaattatgtaaaaagtgtgatttatgccctacaggaagccctcagagtctacctccctaccccagcgtccccccgactccttccccaactaataaggaccccccttcaacccaaacggtccaaaaggagatagacaaaggggtaaacaatgaaccaaagagtgccaatattccccgattatgccccctccaagcagtgggaggaggagaattcggcccagtcagagtgcatgtacctttttccctctcagacttgaagcaaattaaaatagacctaggtaaattctcagataaccctgatggctatattgatgttttacaagggttaggacaatcctttgatctgacatggagagatataatgttactgctagatcagacactaaccccaaatgagagaagtgccgccataactgcagcccgagagtttggcgatctctggtatctcagtcaggtcaatgataggatgacaacagaggaaagagaacaattccccacaggccagcaggcagttcccagtgtagaccctcattgggacgcagaatcagaacatggagattggtgccgcagacatttgctaacttgcgtgctagaaggactaaggaaaactaggaagaagcctatraattattcaatgatgtccactataacacagggaaaggaagaaaatcctactgcctttctggagagactaagggaggcattgaggaagcatacctctctgtcacctgactctattgaaggccaactaatcttaaaggataagtttatcactcagtcagctgcagacattagaaaaaaacttcaaaagtcygccttaggcccggagcaaaacttagaaaccctattgaacttggcaacctcggttttttataatagagatcaggaggagcaggcggaaygggacaaacgggataaaaaaaaggccaccgctttagtcatggccctcaggcaagcggactttggaggctctggaamagggaaaggctgggcaaatcgaatgcctaatagggcttgcttccagtgcggtctacaaggacactttaaaaaagattgtccgaatagaaataagccrccccctcgtccatgccccttatgtcaagggaatcactggaaggcccactgccccaggggaygaaggtcctctgagtcagaagccactaaccagatgatccagcagcaggactgagggtgcccggggcaagcgccagcccatgccatcaccctcacagagccccgggtatgcttgaccattgagggccaggaggttaactgtctcctggacactggcgcggccttctcagtcttactctcctgtcccggacaactgtcctccagatctgtcactatccgaggggtcctaggacaggcagtcactagatacttctcccagccactaagttgtgactggggaactttactcttttcacatgcctttctaattatgcctgaaagccccactcctttgttagggagagacattctagcaaaagcaggggccattatacacctgaacataggagaaggaacacccgtttgttgtcccctacttgaggaaggaattaatcctgaagtctgggcaacagaaggacaatatggaygagcaaagaatgcccgtcctgttcaagttaaactaaaggattccgcctcctttccctaccaaaggcagtacccccttagacccgaggcccaacaaggactccaaaagattgttaaggacctaaaagcccaaggcctagtaaaaccatgcaatagcccctgcaatactccaattttaggagtacagaaacccaatggacagtggaggttagtgcaagatctcaggattatcaatgaggccrttgtccctctatacccagctgtacctaacccttatactctgctttcccaaataccagaggaagcagagtggtttacagtcctggaccttaaggatgcctttttctgcatccctgtacatcctgactctcaattcttgtttgcctttgaagatccttcgaacccaacgtctcaactcacctggactgttttaccccaagggttcagggatagcccccatctatttggccaggcattagcccaagacttgagccagttctcatacttggacactcttgtcctttggtacgtggatgatttacttttagccgcccgttcagaaaccttgtgccatcaagccacccaagcgctcttaaacttcctcgccacctgtggctacaaggtttccaaaccaaaggctcagctctgctcacagcaggttaaatacttagggctaaaattatccaaaggcaccagggccctcagtgaggaatgtatccagcctatactggcttatcctcatcccaaaaccctraagcaattaagagggttccttggcataacaggcttctgccgaatatggattcccaggtacggcgaaatagccaggccattatatacactaattaaggaaactcagaaagccaatacccatttagtaagatggacacctgaagcagaagcggctttccaggccctaaagaaggccctaacccaagccccagtgttaagcttgccaacggggcaagacttttctttatatgtcacagaaaaaacaggaatagctctaggagtccttacacaggtccgagggaccagcttgcaacccgtggcatacctgagtaaggaaattgatgtagtggcaaagggttggcctcattgtttatgggtagtggcagcagtagcagtcttagtatctgaagcagttaaaataatacagggaagagatcttactgtgtggacatctcatgatgtgaacggcatactcactgctaaaggagacttgtggctgtcagacaaccrtttgcttaaatatcaggctctattacttgaagggccagtgctgcgactgcrcacttgtgcaactcttaacccagccacatttcttccagacaatgaagaaaagatagaacataactgtcaacaagtrattgctcaaacctacgccgctcgaggggaccttctagaggttcccttgactgatcccgacctcaacttgtatactgatggaagttcctttgtagaaaaaggacttcgaaaagcggggtatgcagtggtcagtgataatggaatacttgaaagtaatcccctcactccaggaactagcgctcagctggcagaactaatagccctcactcgggcactagaattaggagaaggaaaaagggtaaatatatatacagactctaagtatgcttacctagtcctccatgcccacgcagcaatatggagagaaagggaattcctaacttccgagggaacacctatcaaacatcaggaagccattaggagattattattggctgtacagaaacctaaagaggtggcagtcttacactgccggggtcatcagaaaggaaaggaaagggaaatagaagggaaccgccaagcggatattgaagccaaaagagccgcaaggcgggaccctccattagaaatgcttatagaaggacccctagtatggggtaatcccctccaggaaaccaagccccagtactcagcagaagaaatagaatggggaacctcacgaggacatagtttcctcccctcaggatggctagccaccgaagaaggaaaaatacttttgcctgcagctaaccaatggaaattacttaaaacccttcaccaaacctttcacttaggcattgatagcacccatcagatggccaaatcattatttactggaccaggccttttcaaaactatcaagcagatagtcagggcctgtgaagtgtgccaaagaaataatcccctgccttatcgccaagctccttcaggagaacaaagaacaggccattacccaggagaagactggcaactagattttacccacatgcccaaatctcagggatttcagtatctactagtctgggtagatactttcactggttgggcrgaggccttcccttgtaggacagaaaaggcccaagaggtaataaaggcactaattcatgaaataattcccagattcggacttccccgaggcttacagagtgacaatggccccgctttcaaggctgcagtaacccagggagtatcccaggcgttaggcatacaatatcacttacactgcgcctggaggccacaatcctcaggaaaagtcgagaaaatgaatgaaacactcaaacgacatctaaaaaagctaacccaagaaacccaccttgcatggcctgctctgttgcctatagccttactaagaatccgaaactctccccaaaaagcaggacttagcccatacgaaatgctgtatggacrgcccttcctaaccaatgaccttgtgcttgaccgagagayggccaacttagttgcagacatcacctccttagccaaatatcaacaagttcttaaaacattacagggaacctgtccccgagaggagggaaaggaattattccaccctggtgacatggtattagtcaagtcccttccctctaattccccatccctagatacatcctgggaaggaccctacccagtcattttatctaccccaaccgcggttaaagtggctggagtggagtcttggatacatcacactcgagtcaaaccctggatactgccaaaggaacccgaaaatccaggagacaacgctagctattcctgtgaacctctagaggatctgcgcctgctcttcaagcgacaaccgtgaggaaagtaactagaatcgtagatccccatggccctcccttgtcatatttttctttttactgttctcttaccccctttcactctcactgcacctcctccatgccgctgtactaccagtagctccccttaccaagagcttctatggagaatgcagcttcccagaaatattgatgccccatygtataggagtttttctaaaggaaaccccactttcaccgcccacacccatatgccccacaactgctataactctgccactctttgcatgcatgcaaatactcattattggacagggaaaatrattaatcctagttgtcctggagggcttggagccactgtctgttggacttacttcacccatactggtatgtctgatgggggtggagttcaagatcaggcaagagaaaaacacgtaaaggaagtaatctcccaactgacccgggtacatagcacccctagcccctacaaaggactagacctctcaaaactacatgaaaccctccatacccatactcacctggtaagcctatttaataccaccctcactgggctccatgaagccttggcccaaaaccctactaactgttggatgtgcctccccctgcacttcaggccatacatttcaatccctgtacctgaacaatggaacaacttcagcacagaaataaacaccacttccgttttagtaggacctcttgtttccaatctggaaataacccatacctcaaacctcacctgtgtaaaatttagcaatactatagacacaaccaactcccaatgcatcaggtgggtaactcctcccacacgaatagtctgcctaccctcaggaatattttttgtctgtggtacctcagcctatcgttgtttgaatggctcttcagaatctatgtgcttcctctcattcttagtgccccctatgaccatctacactgaacaagatttatacagttatgtcgtacctaagccccgcaacaaaagagtacccattcttccttttgttatcggagcaggagtgctaggtggactaggtactggcattggcggtatcacaacctctactcagttctactacaaactatctcaagaactaaatggggacatggaacgggtcgccgactccctggtcaccttgcaagatcaacttaactccctagcagcagtagtccttcaaaatcgaagagctttagacttgctaaccgctgaaagagggggaacctgtttatttttaggggaagaatgctgttattatgttaatcaatccggaatcgtcactgagaaagttaaagaaattcgagatcgaatacaacgtagagcagaggagcttcgaaacaccggaccctggggcctcctcagccaatggatgccctggattctccccttcttaggacctctagcagctataatattgctactcctctttgccctgtatctttaacctccttgttaagtttgtctcttccagaatcgaagctgtaaaactacaaatcgttcttcaaatggagccccagatgcagtccatgactaagatctaccgcggacccctggaccggcctgctagcccatgctccgatgttgatgacatcgaaggcacccctcccgaggaaatctcaactgcacgacccctactatgccccaattcagcaggaagcagttagagcggtcgtcggccaacctccccaacagcacttgggttttcctgttgagaggggggac**tgagagacaggactagctggatttcctaggccgactaagaattcctaagcctagctggggaaggtgacyrcacccacctttaaacayggggcttgtaactcagctcacacccgaccaatcaggtagtaaagagrgctcactaaaatacmaattaggctaaaagcaggaggtaaagaaatagtcaaatcatatatcrcctgagagcacagggggagggacaatgatygggatataaacccaggcattcgagcmgggagtggcaaccccctttgggtcccctcccattgtatgggagctctgttttcactctattaaatcttgcaactgcacactcttctggtccgtgtttgttmcggctcragctgagctttcgctcrccgtccaccactgctgtttgccgccgtcgcagacccgccactgacttccacccctcyggatccggcagggtgtccrctgygctyctgatccagygaggcgcccattgccrctccygatcgggctaraggctcrccattgttcctgcatggctaagtgcccgggttcrtcctaatcgagctgaacactagtcgctgggttccacggttctcttccrtgacccacggcttctaatagagctataacactcaccgcatggcccaaggttccattccttggaatccgtgaggccaagaaccccaggtcagagaacaaaaggcttgctgccatcttggragcggcccgccaccatcttgggagctctaagaacaaagacccmccmgtaaca**
